# Parental alcohol use and risk of behavioral and emotional problems in offspring

**DOI:** 10.1371/journal.pone.0178862

**Published:** 2017-06-06

**Authors:** Liam Mahedy, Gemma Hammerton, Alison Teyhan, Alexis C. Edwards, Kenneth S. Kendler, Simon C. Moore, Matthew Hickman, John Macleod, Jon Heron

**Affiliations:** 1 School of Social and Community Medicine, University of Bristol, Bristol, United Kingdom; 2 Department of Psychiatry and School of Medicine, Virginia Institute for Psychiatric and Behavioral Genetics, Virginia Commonwealth University, Richmond, Virginia, United States of America; 3 School of Dentistry, College of Biomedical and Life Science, Cardiff University, Cardiff, United Kingdom; Erasmus University Rotterdam, NETHERLANDS

## Abstract

**Objective:**

The majority of studies that have examined parental alcohol use and offspring outcomes have either focused on exposure in the antenatal period or from clinical populations. This study sought to examine proximal and distal associations between parental alcohol use and offspring conduct problems and depressive symptoms in a population birth cohort.

**Methods:**

We used prospective data from a large UK based population cohort (ALSPAC) to investigate the association between parental alcohol use, measured in units, (assessed at ages 4 and 12 years) with childhood conduct trajectories, (assessed on six occasions from 4 to 13.5 years, *n* = 6,927), and adolescent depressive symptoms (assessed on four occasions from ~13 to ~18 years, *n* = 5,539). Heavy drinking was defined as ≥21 units per week in mothers and partners who drank 4+ units daily.

**Results:**

We found little evidence to support a dose response association between parental alcohol use and offspring outcomes. For example, we found insufficient evidence to support an association between maternal alcohol use at age 4 years and childhood conduct problems (childhood limited: OR = 1.00, 95% CI = .99, 1.01; adolescent onset: OR = 0.99, 95% CI = .98, 1.00; and early-onset persistent: OR = 0.99, 95% CI = .98, 1.00) per 1-unit change in maternal alcohol use compared to those with low levels of conduct problems. We also found insufficient evidence to support an association between maternal alcohol use at age 4 years and adolescent depressive symptoms (intercept: *b* = .001, 95% CI = -.01, .01, and slope: *b* = .003, 95% CI = -.03, .03) per 1-unit change in maternal alcohol use. Results remained consistent across amount of alcohol consumed (i.e., number of alcohol units or heavy alcohol use), parent (maternal self-reports or maternal reports of partner’s alcohol use), and timing of alcohol use (assessed at age 4 or age 12 years).

**Conclusions:**

There is no support for an association between parental alcohol use during childhood and conduct and emotional problems during childhood or adolescence.

## Introduction

An estimated 2.6 million children in the UK (22%) live with a parent who drinks hazardously [1]. Parental alcohol use can have a profound impact on their child’s development, with children showing increased emotional and behavioral difficulties [2]. Alcohol epidemiology has traditionally focused on alcohol exposure during the antenatal period e.g., [3–9], and has largely focused on substance use and educational outcomes [10]. Less is known about the influence of parental alcohol use during childhood and the impact on offspring mental health outcomes. The studies that have examined the association between parental alcohol use during childhood and offspring mental health outcomes have mainly focused on samples involving heavy alcohol use or alcoholic parents and outcomes in very early childhood [11–14]. However, there is a need to examine the association between light to moderate alcohol use and child outcomes across childhood and adolescence.

All studies focusing on alcohol use suffer from some methodological considerations. For example, under-reporting of alcohol consumption is a challenge in observational studies [15] and is particularly common in studies based on pregnancy samples [16]. Furthermore, the paradoxical *protective* effects of antenatal parental alcohol use found in some studies [9,17,18] are most likely explained by 1) misclassification of the exposure or outcome, 2) residual confounding, or 3) small sample size [6,19]. One possible explanation for these *protective* effects in the literature is that offspring outcomes were measured at only one occasion; that is, some of the behaviors being examined may not have been evident at the time of measurement, as there is evidence that problems vary across time [20].

One previous study [21], using this sample (Avon Longitudinal Study of Parents and Children, ALSPAC), examined alcohol use in both the antenatal and postnatal periods, found that maternal alcohol consumption was positively associated with offspring externalizing behaviors. However, these effects were largely evident for maternal alcohol consumption during pregnancy, rather than the postnatal period, suggesting evidence for fetal exposure to the intrauterine effects to alcohol [8].

In this study, we extend previous work, that has used the ALSPAC sample, to examine parental alcohol use in the antenatal period e.g., [6,7,21–23] by focusing on the association between parental alcohol use during childhood and longitudinal trajectories of youth mental health problems. Specifically, the aim is to use a more flexible trajectory approach, allowing for developmental variability, to examine the impact of both proximal and distal measures of parental alcohol use on youth behavioral and emotional problems.

## Methods

### Participants and procedure

We used data from the Avon Longitudinal Study of Parents and Children (ALSPAC), which recruited 14,541 pregnant mothers who resided in the former Avon Health Authority in the southwest of England, and had an estimated date of delivery between April 1, 1991 and December 31, 1992. Of the 13,978 offspring alive at one year, a small number of participants withdrew from the study (*n* = 24), leaving a starting sample of 13,954. ALSPAC provide a range of options for withdrawal of consent e.g., participation break, withdrawal from direct participation, withdrawal from study—maintaining permission to use existing data. For a detailed overview of our study population including attrition at the different measurement occasions (S1a–S1c Fig). Detailed information about ALSPAC is available online www.bris.ac.uk/alspac and in the cohort profiles [24,25]. A fully searchable data dictionary is available on the study’s website (www.bris.ac.uk/alspac/researchers/data-access/data-dictionary/). Ethical approval for the study was obtained from the ALSPAC Ethics and Law Committee and the Local Research Ethics Committees.

### Measures

#### Parental alcohol consumption

We used a combination of maternal reports of their own and their partner’s alcohol consumption. In assessing maternal alcohol use, mothers completed a postal questionnaire at *47 months* and *12 years 1 month* asking about their daily alcohol consumption. From here, we refer to these measurement occasions as age 4 and 12 years respectively. Reponses were converted into units e.g., ½ pint of ‘beer, larger, or cider’ reflects 1 unit, one glass of wine reflects 2 units, one pub measure ‘spirit’ reflects 1 unit, ‘other alcoholic drinks’ reflects 1 unit, and ‘low alcoholic drinks’ reflects 0.5 of a unit; ‘sherry and others’ reflects 1 unit; and ‘ready mix drinks’ reflects 1.5 units. One drink was equivalent to one UK unit of alcohol, corresponding to 8 grams of alcohol. For our analyses maternal alcohol exposure was defined as a total sum score reflecting the number of alcohol units consumed in one week. Heavy alcohol use was defined as drinking ≥21 units per week, reflecting on average 3 units per day, considerably above the weekly governmental guidelines of 14 units of alcohol [26].

We also used a maternal-reported measure of partner alcohol consumption collected at the same measurement occasions. Partner’s frequency of drinking 4+ units was assessed using the following question: ‘How many days in the past month do you think he had the equivalent of 2 pints of beer, 4 glasses of wine or 4 pub measures of spirit?’ Response options were: *none*, *1–2 days*, *3–4 days*, *5–10 days*, *more than 10 days*, and *every day*, treated as a continuous measure. Heavy alcohol use was defined as drinking 4+ units daily.

#### Offspring outcome measures

Maternal reports of youth conduct problems were measured using the conduct subscale of the Strengths and Difficulties Questionnaire (SDQ) [27] assessed on six occasions at approximately 4, 7, 8, 10, 12, and 13.5 years. The SDQ is a well-validated measure with a large amount of peer-reviewed literature detailing its psychometric properties [28,29]. Of the sample of 9,600 mothers who had information on alcohol use, 72.1% (*n* = 6,927/9,600) of offspring had information on at least four measures of conduct problems. There was strong evidence of a relationship between sociodemographic variables and trajectories of conduct problems (S1 Table).

Heterogeneity in childhood conduct problems is well established, see [30,31]. From this framework, Barker and Maughan [32] derived four developmental trajectories of conduct problems throughout childhood using latent class growth analysis which have been used in a number of subsequent analyses e.g., [33–35]. Sum scores were dichotomised at the threshold of four or more at each measurement occasion. Binary cut-offs were established based on national norms established for children in England and Wales [36]. The four-class model comprised of children with low involvement with conduct problems (Low, 64% of the sample, 48.9% boys), childhood limited (CL, 15% of the sample, 54.1% boys), adolescent onset (AO, 12% of the sample, 49.7% boys), and early onset persistent (EOP, 9% of the sample, 56.8% boys). Focusing on these conduct trajectory groupings allows for developmentally sensitive patterns of conduct problems to be examined, rather than the approach of focusing on one time point, which assigns similarity to children who display similar levels of problems at this time point but importantly display different developmental trajectories. For our purposes, we examined trajectories of conduct problems for the sample as a whole as the previous work by Barker and Maughan found that gender-invariant models provided adequate fit to the data.

Adolescent depressive symptoms were measured using the self-report Mood and Feelings Questionnaire—short form (MFQ) [37] assessed on four occasions at 12y 10m, 13y 10m, 16y 6m, and 17y 10m. Of the sample of 9,600 mothers who had information on alcohol use, 57.7% (*n* = 5,539) of offspring provided at least two measures of depressive symptoms. There was strong evidence of a relationship between sociodemographic variables and missing data on depressive symptoms (S2 Table). Previously, Edwards and colleagues [38] using a dimensional approach, found strong evidence of variation in symptom levels at baseline and also symptom growth for males and females respectively.

For the purposes of our study, we estimated latent growth models for males and females separately using the *knownclass* option in M*plus* as the development of depressive symptoms has previously been shown to differ across gender during early adolescence e.g., [39]. As well as maximizing power, this approach produces a single covariate estimate by constraining the association between the covariate with the intercept and slope to be equal and by constraining the variances to be equal. The residual variances were freely estimated across gender, but constrained within time.

#### Potential confounding variables

A range of measures were considered to be potential confounders of the parental alcohol—offspring mental health relationship. These comprised of established risk factors for conduct problems and depression outcomes for which we felt the assumption of a causal predictive relationship with parental alcohol use could be justified. Measures of social economic position (SEP) were recorded by maternal self-report questionnaires during pregnancy. These included maternal age at delivery, parity (1, 2, ≥3 children), socioeconomic position (grouped into four categories: 1) unskilled/semiskilled manual; 2) skilled manual/nonmanual; 3) managerial/technical; and 4) professional), maternal education (<O level: indicating no qualification; O level: indicating completion of school examinations at age 16; and >O level: indicating completion of college or university education at or after age 18), maternal smoking during first trimester in pregnancy (yes/no), housing tenure (mortgaged, subsidised renting, private renting), income (measured in quintiles), and maternal depressive symptoms measured using the Edinburgh Postnatal Depression Scale [40] at 32 weeks gestation

### Statistical methods

#### Childhood conduct problems

The association between parental alcohol consumption at age 4 years (using linear and binary exposures) and developmental trajectories of offspring conduct problems across all 6 timepoints (i.e., EOP, AO, CL, and Low as the reference group) were examined using multinomial logistic regression. In estimating class membership, simulation work [41–43] demonstrated that the standard three-step modal class approach can introduce bias affecting the strength of the association between the latent classes and observed covariates. For this reason, we used the ‘auxiliary (r3step)’ command in M*plus*, which allows for bias-adjusted estimates and has been shown to lead to less-biased estimates than the traditional three-step methods [44]. This approach allows for the most likely class membership to be obtained from the posterior probabilities along with classification uncertainty; the most likely class membership variables can then be analyzed to include covariates while accounting for the measurement error in classification [45].

#### Adolescent depressive symptoms

Offspring depressive symptom scores across all four time points were used. The intercept factor loadings were all fixed at one and the slope factor loadings were fixed to reflect the amount of time in months between assessments with baseline at zero. After the unconditional models were estimated, a series of four separate conditional latent growth models (LGM), which included maternal and partner alcohol consumption at age 4 years (i.e., distal exposure) and at age 12 years (i.e., proximal exposure) as predictors of depressive symptoms intercept and slope factors, at each time point were estimated.

### Missing data

9,600 mothers provided self-report information on alcohol use at age 4 years. Of these, 8,139/9,600 (84.8%) provided information on their partners alcohol use at the same time period. At age 12 years, 5,931/9,600 (61.8%) mothers provided self-report information on alcohol use. Of these, 5,535/9,600 (57.7%) provided information on their partner’s alcohol use at the same time period. Since all of the confounders were assessed in early pregnancy, there was minimal missing data (e.g., parental social class had the most amount of missing data: 817/9,600 (8.5%)). Associations between background socioeconomic variables and offspring conduct problems and depressive symptoms are presented in S3 and S4 Tables.

Using complete case analysis and not taking missing data into account can result in biased estimates [46]. As previously described [32,38], missing data on the outcome measures were dealt with using full information maximum likelihood (FIML). Once the outcome models were derived, inverse probability weighting (IPW) [47] was used as a sampling weight to investigate the possible influence of selective participation on our estimates of association between parental alcohol and offspring conduct problems and depressive symptoms, respectively. In this way, estimates were weighted to account for probabilities of nonresponse for the mental health outcomes. The process of weighting, using IPW, allows us to give more weights to individuals who have similar prenatal characteristics to those of individuals who are likely to subsequently be lost to the study.

Within the sample with complete data on maternal alcohol, IPW was used to derive logistic regression models predicting having complete conduct problem trajectory data (*n* = 6,927). Response rates differed according to: maternal age, offspring gender, grandmother having a history of severe depression, maternal alcohol use in pregnancy, intentional pregnancy, damp/mould on walls in the house, marital status, and car ownership (S5 Table). The Hosmer-Lemeshow test was used to assess model fit and included respondents were then weighted by the inverse of this probability. The reciprocal of the predicted probabilities from these models were used as sampling weights to adjust the regression models of interest. IPW was performed using Stata 13. The weighted models are presented as our main findings; the unweighted results are presented in S6a–S7b Tables.

This procedure was repeated for the depressive symptoms model; logistic regression model predicting having at least two measures of depressive symptoms (*n* = 5,539). Of these, *n* = 1,624 had information on three measures; while *n* = 2,422 had information on all four depression measures. Again there was evidence of an association between all of the variables with loss to follow-up (results available on request). Weights ranged from 1.2 to 18.4 for conduct problems models and from 1.3 to 21.4 for depression models. Due to the potential for extreme weighted values adversely influencing subsequent analyses, larger weights were trimmed to 10. All models were analysed in M*plus* v7.11 using the maximum likelihood estimator [48].

## Results

### Descriptive results

Available information for maternal alcohol use and partner’s frequency of drinking 4+ units at ages 4 and 12 years and conduct problems and depressive symptoms is reported in Table 1. There was good agreement between maternal and partners reports of partners frequency of drinking 4+ units at 4 years (ĸ = .87).

**Table 1:** Descriptive information for maternal and partner alcohol use for both conduct problems and depressive symptoms.

	Conduct problems trajectories sample	Depressive symptoms trajectories sample
**Age 4 years**	M (SD)	M (SD)
**Maternal alcohol use in units—linear term**	7.50 (9.5)	7.68 (9.5)
**Maternal drinking ≥21 units**	*n* (%)	*n* (%)
	605/6,927 (8.7)	502/5,539 (9.1)
**Partner drinking 4+ units**^1^ **- linear term**	*n* (%)	*n* (%)
None	984/6,063 (16.2)	788/4,887 (16.1)
1–2 days	1,087 (17.9)	899 (18.4)
3–4 days	1,226 (20.2)	981 (20.1)
5–10 days	1,489 (24.6)	1,218 (24.9)
>10 days	957 (15.8)	744 (15.2)
Everyday	320 (5.3)	257 (5.3)
**Age 12 years**		M (SD)
**Maternal alcohol use in units—linear term**	----	10.78 (11.8)
**Maternal drinking ≥21 units**	----	*n* (%)
		718/4,818 (14.9)
		*n* (%)
**Partner drinking 4+ units**^1^ **- linear term**	----	4,549
None		852 (18.7)
1–2 days		830 (18.3)
3–4 days		785 (17.3)
5–10 days		966 (21.3)
>10 days		781 (17.2)
Everyday		335 (7.4)

Note:

^1^Maternal reports of partner’s alcohol consumption

Table 2 shows the association between the parental alcohol measures. We found strong evidence of an association between alcohol measures for each parent within and across time, with stronger associations within person and within time.

**Table 2:** Correlations between parental alcohol measures (linear term) at age 4 and 12 years.

	1	2	3	4
**Age 4 years**				
**1.** Maternal alcohol use in units	1			
**2.** Partner frequency drinking 4+ units^1^	.315	1		
**Age 12 years**				
**3.** Maternal alcohol use in units	.519	.263	1	
**4.** Partner frequency drinking 4+ units^1^	.248	.577	.348	1

Note:

^1^Maternal reports of partner’s alcohol consumption

#### Group-based trajectories of conduct problems

Table 3 presents the associations between parental alcohol use at age 4 years (using linear and binary terms) and the four conduct trajectory classes. The pattern of results did not change after controlling for confounding variables. Overall, using the weighted estimates, we found insufficient evidence of an association between parental alcohol use at age 4 years and group-based trajectories of conduct problems across childhood.

**Table 3:** Parental alcohol consumption (assessed at age 4 years using linear and binary measures) and childhood conduct problem trajectories.

	Unadjusted models					Adjusted models^2^				
	*N*	CL OR (95% CI)	AO OR (95% CI)	EOP OR (95% CI)	*p*	*N*	CL OR (95% CI)	AO OR (95% CI)	EOP OR (95% CI)	*p*
**Linear alcohol measure**										
Maternal drinking	6,927	1.00 (.99, 1.01)	0.99 (.98, 1.00)	0.99 (.98, 1.00)	.42	6,014	1.00 (.99, 1.01)	0.99 (.98, 1.00)	0.99 (.98, 1.00)	.48
Partner drinking^1^	6,063	0.97 (.84, 1.12)	0.95 (.86, 1.06)	0.92 (.80, 1.05)	.81	5,359	1.04 (.94, 1.14)	1.01 (.90, 1.14)	0.97 (.89, 1.06)	.83
**Binary alcohol measure**										
Maternal drinking (8.7%)	6,927	1.56 (1.05, 2.34)	0.71 (.34, 1.45)	0.99 (.64, 1.52)	.17	6,014	1.40 (.93, 2.11)	0.61 (.30, 1.24)	0.76 (.48, 1.20)	.22
Partner drinking^1^ (5.2%)	6,063	0.77 (.39, 1.51)	0.94 (.44, 1.98)	1.45 (.94, 2.25)	.27	5,359	0.81 (.42, 1.57)	0.73 (.26, 2.01)	1.26 (.79, 2.02)	.58

Note:

^1^Maternal reports of partner’s alcohol consumption;

^2^Multinomial logistic regression models adjusted for maternal age at delivery, parity, SEP, maternal education, maternal smoking during first trimester in pregnancy, housing tenure, income, and maternal depressive symptoms at 32 weeks gestation;

CL: childhood limited, AO: adolescent onset, EOP: early onset persistent, the Low conduct problems class was used as the reference group.

There was a suggestion of an association between maternal alcohol use examining heavy parental alcohol use and conduct problems limited to childhood compared to low conduct problems (OR = 1.56, 95% CI = 1.05, 2.34). However, this association was attenuated when models were adjusted for confounding variables (OR = 1.40, 95% CI = 0.93, 2.11). We found little evidence for any further associations.

#### Trajectories of adolescent depressive symptoms

Table 4 presents the associations between parental alcohol use at ages 4 and 12 years (using linear and binary terms) and adolescent depressive symptoms before and after adjusting for potential confounding variables. There was weak evidence of an association between partner alcohol use and baseline adolescent depressive symptoms after adjusting for confounding variables (*b* = -.065, 95% CI = -.13, -.00, *p* = .05). We found little evidence of an association between heavy parental alcohol use (using binary alcohol exposures) for either unadjusted or adjusted models. Results highlighting the pattern of depressive symptoms across adolescence for males and females grouped by heavy and non-heavy maternal alcohol use at age 4 years are displayed in the Supplementary Material (S2 Fig). Finally, as a sensitivity set of analyses, antenatal alcohol use at age 18 weeks gestation was included (S8a–S9b Tables). The pattern of results remain unchanged.

**Table 4:** Parental alcohol use (assessed at age 4 and 12 years using linear alcohol measures) and adolescent depressive symptoms.

	Unadjusted models					Adjusted models^2^				
	*N*	Intercept *b* (95% CI)	*p*	Slope *b* (95% CI)	*p*	*N*	Intercept *b* (95% CI)	*p*	Slope *b* (95% CI)	*p*
**Linear alcohol measure**										
**Age 4 years**										
Maternal drinking	5,539	.004 (-.01, .01)	.41	-.011 (-.04, .01)	.38	4,837	.001 (-.01, .01)	.84	.003 (-.03, .03)	.86
Partner drinking	4,887	-.036 (-.10, .02)	.24	.056 (-.12, .23)	.53	4,335	-.065 (-.13, -.00)	.05	.074 (-.11, .26)	.43
**Age 12 years**										
Maternal alcohol	4,818	.005 (-.00, .01)	.21	.010 (-.01, .03)	.37	4,133	.001 (-.01, .01)	.77	.019 (-.01, .04)	.12
Partner drinking	4,549	.001 (-.06, .06)	.97	-.021 (-.19, .15)	.81	3,901	-.043 (-.11, .02)	.17	.003 (-.18, .18)	.97
**Binary alcohol measure**										
**Age 4 years**										
Maternal drinking (9.1%)	5,539	.164 (-.11, .44)	.25	.031 (-.81, .88)	.94	4,837	.024 (-.25, .30)	.86	.264 (-.58, 1.11)	.54
Partner drinking (5.2%)	4,887	-.026 (-.40, .35)	.89	.323 (-.99, 1.64)	.63	4,335	-.147 (-.55, .26)	.48	.784 (-.63, 2.20)	.28
**Age 12 years**										
Maternal drinking (14.9%)	4,818	.040 (-.20, .28)	.74	.457 (-.26, 1.18)	.21	4,133	-.004 (-.24, .24)	.97	.636 (-.11, 1.38)	.09
Partner drinking (7.4%)	4,549	.329 (-.04, .71)	.08	.710 (-.28, 1.70)	.16	3,901	-.068 (-.44, .31)	.72	.968 (-.05, 1.99)	.06

Note:

^1^Maternal reports of partner’s alcohol consumption;

^2^Models adjusted for maternal age at delivery, parity, social economic position, maternal education, maternal smoking during first trimester in pregnancy, housing tenure, income, and maternal depressive symptoms at 32 weeks gestation

## Discussion

Based on a large prospective birth cohort, we found insufficient evidence of an association between parental alcohol, measured using proximal and distal measures, and trajectories of childhood conduct problems or adolescent depressive symptoms. Although there was a suggestion of some weak associations using the unadjusted models, these associations were attenuated when models were adjusted for a number of confounding variables.

The present study should be considered in light of a number of limitations. As with any longitudinal study, data were not complete on exposures, outcomes, and confounders for the whole cohort. Although we cannot rule out the possibility that exclusion of mothers without complete information might have biased our findings for reported alcohol use, there was minimal change to the models when differential dropout was accounted for using inverse probability weighting. Furthermore, a previous study found evidence that differential attrition does not affect estimates of risk for behavioural disorders in ALSPAC [49]. Second, self-reported alcohol information is generally underreported, however there is evidence that reports outside of the antenatal period may be more accurate than prenatal reports given that alcohol use in pregnancy is underreported to a greater extent [16]. Third, although the assessment of parental alcohol use was different in terms of quantity and frequency of alcohol consumed, they do capture a broad account of parental drinking practices. Fourth, the use of partner reports of their own alcohol usage would have been optimal, however maternal reports of their partners alcohol use were used because of 1) the rate of attrition in partner reporting, especially for the assessment at age 12 years, and 2) the very good inter-rater agreement between parental reports of partners drinking practices. Fifth, it is possible that the association between parental alcohol use and offspring outcomes is only evident at the extreme end of alcohol consumption. In examining this possibility, we found no evidence to support an association between *heavy* alcohol use (using binary measures of maternal and partner’s alcohol use) and offspring conduct and emotional problems. Sixth, one of our outcomes (group-based trajectories of conduct problems) was based on maternal reports which raises the possibility of measurement bias due to differential misclassification of the outcome (i.e., conduct problems) across exposure groups (alcohol use versus no alcohol use). Finally, although the SDQ is used as a screening instrument for mental health problems in epidemiological research rather than providing a clinical diagnoses, a recent systematic review [50] reported that parent-reported versions of the conduct problems subscale had a sensitivity of 0.75 and a specificity of 0.91 for the detections of conduct disorder.

### Comparison with previous studies

Unlike the few studies that have examined the association between parental alcohol use during childhood and offspring emotional and behavioral outcomes [51,52], we found little evidence in support of this association. The contrast in findings could be due to a number of possibilities. First, our study used a population sample in contrast to the majority of previous studies that have used clinical samples. For example, the studies by Hussong and colleagues defined their alcohol measure as a lifetime diagnosis of alcoholism, while in our study, alcohol use largely reflected more light to moderate drinking practices. The use of clinical samples may lead to an overestimation of the association between parental alcohol use and youth outcomes due to the selection of more severely impaired parents, therefore limiting generalizability [53]. Furthermore, large population samples are better powered to detect associations compared to smaller clinical samples, as the use of relatively small samples may indicate that bias could be impacting on the findings. Second, our study utilized more robust outcome measures (i.e., group-based trajectory modelling and latent growth models), capturing rich longitudinal information over childhood and adolescence which is important for following markedly different developmental trajectories.

Third, being able to examine maternal and paternal alcohol use separately is important as previous studies have suggested that maternal alcohol use may be more hazardous to offspring mental health problems than paternal alcohol use [53–55]. On this note, there was no evidence of an association between parental alcohol consumption and offspring conduct problems, as these symptoms show stronger relations compared to emotional symptoms [55]. Finally, we found little evidence of an association between distal or proximal effects of parental alcohol use on youth mental health problems which have previously been shown in children of alcoholics to be largely related to distal factors for both externalizing and internalizing symptoms [51,52].

Previous studies examining varying levels of antenatal alcohol use (i.e., light, moderate, heavy, and alcohol dependence) have demonstrated adverse effects on a range of offspring outcomes [56]; while others have demonstrated no associations [17,18,57,58]. Although this study was primarily addressing whether parental alcohol use during childhood was associated with later offspring emotional and behavioural difficulties, it is possible that maternal drinking in pregnancy could impact on this association through an intrauterine mechanism, although the findings are mixed. To address this concern, maternal drinking in pregnancy was included as a potential confounder. The overall pattern of results remained unchanged, indicating that light to moderate maternal drinking in pregnancy did not impact on this association. Furthermore, studies that have incorporated a genetic approach to the understanding of the association between maternal alcohol use in the antenatal period and offspring outcomes, using a Mendelian Randomization (MR) design, have on the whole demonstrated adverse associations of moderate maternal drinking in pregnancy and offspring outcomes [5,9,59]. An MR approach was not used in these analyses as the same genetic instruments would be related to the prenatal and postnatal periods and as a result it would not be possible to separate out these effects.

### Implications and conclusions

We found insufficient evidence of an association between parental alcohol use and offspring conduct problems or depressive symptoms—further contributing to the inconsistency of the evidence base on the importance of parental alcohol use during childhood as an influence and risk for offspring mental health outcomes across childhood and adolescence. As our study focused largely on light to moderate parental alcohol use, it cannot be ruled out that findings would differ when examining much higher levels (e.g., alcohol dependence).

## Supporting information

S1 Fig **(A) Flow chart for data availability.** Flowchart showing available data for trajectories of childhood conduct problems and partner alcohol consumption at age 4 years. **(B). Flow chart for data availability.** Flowchart showing available data for adolescent depressive symptoms and partner alcohol consumption at age 4 years. **(C). Flow chart for data availability.** Flowchart showing available data for adolescent depressive symptoms and partner alcohol consumption at age 12 years.
(PDF)

S2 Fig Estimated trajectories of depressive symptoms across adolescence, grouped by heavy and non-heavy parental alcohol use at age 4 years, for maternal alcohol use for males (panel A) and females (panel B), and for partner alcohol use for males (panel C) and females (panel D), with at least two waves of data. (PDF)

S1 Table Descriptive data for key sociodemographic variables–conduct problems. (DOCX)

S2 Table Descriptive data for key sociodemographic variables–depressive symptoms. (DOCX)

S3 Table Univariable associations between demographic variables and trajectories of conduct problems. Note: EOP: early onset persistent, CL: childhood limited, AO: Adolescent onset, the Low group was used as the reference group; SEP: social economic position was grouped into 4 categories: 1: unskilled or semiskilled manual; 2: skilled manual or nonmanual; 3: managerial and technical; and 4: professional.
(DOCX)

S4 Table Univariable associations between demographic variables and offspring depressive symptoms. Note: SEP: social economic position was grouped into 4 categories: 1: unskilled or semiskilled manual; 2: skilled manual or nonmanual; 3: managerial and technical; and 4: professional.
(DOCX)

S5 Table Selective attrition for childhood conduct problems and adolescent depressive symptoms. (DOCX)

S6 Table **(A) Childhood conduct problem trajectories and parental alcohol consumption–unweighted estimates (low group–reference group).** Note: ^1^Maternal reports of partner’s alcohol consumption; ^2^Univariable multinomial logistic regression models; ^3^Multinomial logistic regression models adjusted for maternal age at delivery, parity, Social economic position, maternal education, maternal smoking during first trimester in pregnancy, housing tenure, income, and maternal depressive symptoms at 32 weeks gestation; CL: childhood limited, AO: adolescent onset, EOP: early onset persistent, the Low conduct problems class was used as the reference group. **(B). Heavy parental alcohol consumption (assessed at age 4 years using binary alcohol measures) and childhood conduct problem trajectories–unweighted estimates.** Note: ^1^Maternal reports of partner’s alcohol consumption; CL: childhood limited, AO: adolescent onset, EOP: early onset persistent, the Low conduct problems class was used as the reference group. ^2^Models adjusted for maternal age at delivery, parity, social economic position, maternal education, maternal smoking during first trimester in pregnancy, housing tenure, income, and maternal depressive symptoms at 32 weeks gestation.
(DOCX)

S7 Table **(A) Parental alcohol use (assessed at age 4 and 12 years using linear alcohol measures) and adolescent depressive symptoms–unweighted estimates.** Note. ^1^Maternal reports of partner’s alcohol consumption; ^2^Univariable linear regression models; ^3^Models adjusted for maternal age at delivery, parity, social economic position, maternal education, maternal smoking during first trimester in pregnancy, housing tenure, income, and maternal depressive symptoms at 32 weeks gestation. **(B). Heavy parental alcohol use (assessed at ages 4 and 12 years using binary alcohol measures) and adolescent offspring depressive symptoms–unweighted estimates.** Note: ^1^Maternal reports of partner’s alcohol consumption; ^2^Univariable linear regression models; ^3^Models adjusted for maternal age at delivery, parity, social economic position, maternal education, maternal smoking during first trimester in pregnancy, housing tenure, income, and maternal depressive symptoms at 32 weeks gestation.
(DOCX)

S8 Table **(A) Childhood conduct problem trajectories and parental alcohol consumption–unweighted estimates (low group–reference group).** Note: ^1^Maternal reports of partner’s alcohol consumption; Model 1 adjusted for maternal age at delivery, parity, social economic position, maternal education, maternal smoking during first trimester in pregnancy, housing tenure, income, and maternal depressive symptoms at 32 weeks gestation; Model 2 further adjusted for maternal alcohol use at 18 weeks gestation; CL: childhood limited, AO: adolescent onset, EOP: early onset persistent, the Low conduct problems class was used as the reference group. **(B). Heavy parental alcohol consumption (assessed at age 4 years using binary alcohol measures) and childhood conduct problem trajectories–unweighted estimates.** Note: ^1^Maternal reports of partner’s alcohol consumption; Model 1 adjusted for maternal age at delivery, parity, social economic position, maternal education, maternal smoking during first trimester in pregnancy, housing tenure, income, and maternal depressive symptoms at 32 weeks gestation; Model 2 further adjusted for maternal alcohol use at 18 weeks gestation; CL: childhood limited, AO: adolescent onset, EOP: early onset persistent, the Low conduct problems class was used as the reference group.
(DOCX)

S9 Table **(A) Parental alcohol use (assessed at age 4 and 12 years using linear alcohol measures) and adolescent depressive symptoms–unweighted estimates-complete cases.** Note. ^1^Maternal reports of partner’s alcohol consumption; Model 1 adjusted for maternal age at delivery, parity, social economic position, maternal education, maternal smoking during first trimester in pregnancy, housing tenure, income, and maternal depressive symptoms at 32 weeks gestation; Model 2 further adjusted for maternal alcohol use at 18 weeks gestation. **(B). Heavy parental alcohol use (assessed at ages 4 and 12 years using binary alcohol measures) and adolescent offspring depressive symptoms–unweighted estimates.** Note. ^1^Maternal reports of partner’s alcohol consumption; Model 1 adjusted for maternal age at delivery, parity, social economic position, maternal education, maternal smoking during first trimester in pregnancy, housing tenure, income, and maternal depressive symptoms at 32 weeks gestation; Model 2 further adjusted for maternal alcohol use at 18 weeks gestation.
(DOCX)

## References

[1] Manning V, Best DW, Faulkner N, Titherington E. New estimates of the number of children living with substance misusing parents: results from UK national household surveys. BMC Public Health. 2009;9:377 10.1186/1471-2458-9-377 19814787 PMC2762991

[2] Niccols A, Milligan K, Smith A, Sword W, Thabane L, Henderson J. Integrated programs for mothers with substance abuse issues and their children: A systematic review of studies reporting on child outcomes. Child Abus Negl. 2012;36(4):308–22. 10.1016/j.chiabu.2011.10.007 22483158

[3] Bailey BN, Delaney-Black V, Covington CY, Ager J, Janisse J, Hannigan JH, et al Prenatal exposure to binge drinking and cognitive and behavioral outcomes at age 7 years. Am J Obstet Gynecol. 2004;191(3):1037–43. 10.1016/j.ajog.2004.05.048 15467586

[4] D’Onofrio BM, Van Hulle CA, Waldman ID, Rodgers JL, Rathouz PJ, Lahey BB. Causal inferences regarding prenatal alcohol exposure and childhood externalizing problems. Arch Gen Psychiatry. 2007;64(11):1296–304. 10.1001/archpsyc.64.11.1296 17984398

[5] Murray J, Burgess S, Zuccolo L, Hickman M, Gray R, Lewis SJ. Moderate alcohol drinking in pregnancy increases risk for children’s persistent conduct problems: causal effects in a Mendelian randomisation study. J Child Psychol Psychiatry. 2016;57(5):575–84. 10.1111/jcpp.12486 26588883 PMC4855628

[6] Sayal K, Heron J, Golding J, Emond A. Prenatal alcohol exposure and gender differences in childhood mental health problems: a longitudinal population-based study. Pediatrics. 2007;119(2):e426–34. 10.1542/peds.2006-1840 17272604

[7] Sayal K, Heron J, Draper E, Alati R, Lewis SJ, Fraser R, et al Prenatal exposure to binge pattern of alcohol consumption: mental health and learning outcomes at age 11. Eur Child Adolesc Psychiatry. 2014;23(10):891–9. 10.1007/s00787-014-0599-7 25209690 PMC4186965

[8] Sood B, Delaney-Black V, Covington C, Nordstrom-Klee B, Ager J, Templin T, et al Prenatal alcohol exposure and childhood behavior at age 6 to 7 years: I. dose-response effect. Pediatrics. 2001;108(2):E34 11483844 10.1542/peds.108.2.e34

[9] Zuccolo L, Lewis SJ, Smith GD, Saya K, Draper ES, Fraser R, et al Prenatal alcohol exposure and offspring cognition and school performance. A mendelian randomization natural experiment. Int J Epidemiol. 2013;42(5):1358–70. 10.1093/ije/dyt172 24065783 PMC3807618

[10] Rossow I, Keating P, Felix L, Mccambridge J. Does parental drinking influence children’s drinking? A systematic review of prospective cohort studies. Addiction. 2015;204–17. 10.1111/add.13097 PMC4832292 26283063

[11] Edwards EP, Eiden RD, Colder C, Leonard KE. The development of aggression in 18 to 48 month old children of alcoholic parents. J Abnorm Child Psychol. 2006;34(3): 409–23. 10.1007/s10802-006-9021-3 16649002 PMC2666196

[12] Eiden RD, Edwards EP, Leonard KE. A conceptual model for the development of externalizing behavior problems among kindergarten children of alcoholic families. Dev Psychol. 2007;43(5):1187–201. 10.1037/0012-1649.43.5.1187 17723044 PMC2720575

[13] Keller PS, Cummings EM, Davies PT, Mitchell PM. Longitudinal relations between parental drinking problems, family functioning, and child adjustment. Dev Psychopathol. 2008;20(1):195–212. 10.1017/S0954579408000096 18211734

[14] Knudsen AK, Ystrom E, Skogen JC, Torgersen L. Maternal heavy alcohol use and toddler behavior problems: a fixed effects regression analysis. Eur Child Adolesc Psychiatry. 2015;24(10):1269–77. 10.1007/s00787-015-0677-5 25586409 PMC4592488

[15] Stockwell T, Donath S, Cooper-Stanbury M, Chikritzhs T, Catalano P, Mateo C. Under-reporting of alcohol consumption in household surveys: a comparison of quantity-frequency, graduated-frequency and recent recall. Addiction. 2004;99(8): 1024–33. 10.1111/j.1360-0443.2004.00815.x 15265099

[16] Alvik A, Haldorsen T, Groholt B, Lindemann R. Alcohol consumption before and during pregnancy comparing concurrent and retrospective reports. Alcohol Clin Exp Res. 2006;30(3):510–5. 10.1111/j.1530-0277.2006.00055.x 16499492

[17] Kelly Y, Sacker A, Gray R, Kelly J, Wolke D, Quigley M A. Light drinking in pregnancy, a risk for behavioural problems and cognitive deficits at 3 years of age? Int J Epidemiol. 2009;38(1):129–40. 10.1093/ije/dyn230 18974425

[18] Robinson M, Oddy WH, McLean NJ, Jacoby P, Pennell CE, De Klerk NH, et al Low-moderate prenatal alcohol exposure and risk to child behavioural development: a prospective cohort study. BJOG An Int J Obstet Gynaecol. 2010;117(9):1139–50. 10.1111/j.1471-0528.2010.02596.x 20528867

[19] O’Leary CM, Bower C. Guidelines for pregnancy: what’s an acceptable risk, and how is the evidence (finally) shaping up? Drug Alcohol Rev. 2012;31(2):170–83. 10.1111/j.1465-3362.2011.00331.x 21955332

[20] Moffitt TE, Caspi A, Dickson N, Silva P, Stanton W. Childhood-onset versus adolescent-onset antisocial conduct problems in males: Natural history from ages 3 to 18 years. Dev Psychopathol. 1996;8(2):399–424.

[21] Kendler KS, Gardner CO, Edwards A, Hickman M, Heron J, Macleod J, et al Dimensions of parental alcohol use/problems and offspring temperament, externalizing behaviors, and alcohol use/problems. Alcohol Clin Exp Res. 2013;37(12):2118–27. 10.1111/acer.12196 23895510 PMC3855174

[22] Alati R, Davey Smith G, Lewis SJ, Sayal K, Draper ES, Golding J, et al Effect of prenatal alcohol exposure on childhood academic outcomes: contrasting maternal and paternal associations in the ALSPAC study. PLoS One. 2013;8(10):e74844 10.1371/journal.pone.0074844 24130672 PMC3794033

[23] Sayal K, Heron J, Golding J, Alati R, Smith GD, Gray R, et al Binge pattern of alcohol consumption during pregnancy and childhood mental health outcomes: longitudinal population-based study. Pediatrics. 2009;123(2):e289–96. 10.1542/peds.2008-1861 19171582 PMC6485410

[24] Boyd A, Golding J, Macleod J, Lawlor DA, Fraser A, Henderson J, et al Cohort profile: The “Children of the 90s”-The index offspring of the avon longitudinal study of parents and children. Int J Epidemiol. 2013;42(1):111–27. 10.1093/ije/dys064 22507743 PMC3600618

[25] Fraser A, Macdonald-Wallis C, Tilling K, Boyd A, Golding J, Davey Smith G, et al Cohort Profile: The Avon Longitudinal Study of Parents and Children: ALSPAC mothers cohort. Int J Epidemiol. 2013;42(1):97–110. 10.1093/ije/dys066 22507742 PMC3600619

[26] Department of Health. UK Chief Medical Officers’ alcohol guidelines review: Summary of the proposed new guidelines. 2016;1–7. https://www.gov.uk/government/uploads/system/uploads/attachment_data/file/489795/summary.pdf

[27] Goodman R. The strengths and difficulties questionnaire: a research note. J Child Psychol Psychiatry. 1997;38(5):581–6. 9255702 10.1111/j.1469-7610.1997.tb01545.x

[28] Goodman R. Psychometric properties of the strengths and difficulties questionnaire. J Am Acad Child Adolesc Psychiatry. 2001;40(11):1337–45. 10.1097/00004583-200111000-00015 11699809

[29] Goodman R, Meltzer H, Bailey V. The Strengths and Difficulties Questionnaire: a pilot study on the validity of the self-report version. Eur Child Adolesc Psychiatry. 1998;7(3):125–30. 9826298 10.1007/s007870050057

[30] Moffitt TE. Life-course-persistent versus adolescence-limited antisocial behavior In: Cohen DCDJ, editor. Developmental psychopathology: Risk, disorder, and adaptation, Vol 3, 2nd ed Hoboken, NJ, US: John Wiley & Sons Inc; 2006 p. 570–98.

[31] Moffitt TE, Arseneault L, Jaffee SR, Kim-cohen J, Karestan C, Odgers CL, et al Research Review: DSM-V conduct disorder: research needs for an evidence base. J Child Psychol Psychiatry. 2008;49(1):1–42. 10.1111/j.1469-7610.2007.01823.x PMC2822647 18181878

[32] Barker ED, Maughan B. Limited conduct problem youth. Am J Psychiatry. 2009;166(8):900–8. 10.1176/appi.ajp.2009.08121770 19570930

[33] Gage SH, Hickman M, Heron J, Munafò MR, Lewis G, Macleod J, et al Associations of cannabis and cigarette use with psychotic experiences at age 18: findings from the Avon Longitudinal Study of Parents and Children. Psychol Med. 2014; 44(16):3435–3444. 10.1017/S0033291714000531 25066001 PMC4255321

[34] Kretschmer T, Hickman M, Doerner R, Emond A, Lewis G, Macleod J, et al Outcomes of childhood conduct problem trajectories in early adulthood: findings from the ALSPAC study. Eur Child Adolesc Psychiatry. 2014;23(7):539–49. 10.1007/s00787-013-0488-5 24197169 PMC4172989

[35] Stringaris A, Lewis G, Maughan B. Developmental pathways from childhood conduct problems to early adult depression: findings from the ALSPAC cohort. Br J Psychiatry. 2014;205(1):17–23. 10.1192/bjp.bp.113.134221 24764545 PMC4076653

[36] Meltzer H, Gatward R, Goodman R, Ford T. Mental health of children and adolescents in Great Britain. Int Rev Psychiatry. 2003;15(1–2):185–7. 10.1080/0954026021000046155 12745331

[37] Angold A, Costello EJ, Messer SC, Pickles A, Winder F, Silver D. Developemnt of a short questionnaire for use in epidemiological studies of depression in children and adolescents. Int J Methods Psychaitric Res. 1995;5:237–49.

[38] Edwards AC, Joinson C, Dick DM, Kendler KS, Macleod J, Munafò M, et al The association between depressive symptoms from early to late adolescence and later use and harmful use of alcohol. Eur Child Adolesc Psychiatry. 2014;23(12):1219–30. 10.1007/s00787-014-0600-5 25130265 PMC4246124

[39] Nolen-Hoeksema S, Girgus JS. The emergence of gender differences in depression during adolescence. Psychol Bull. 1994;115(3):424–43. 8016286 10.1037/0033-2909.115.3.424

[40] Cox JL, Holden JM, Sagovsky R. Detection of postnatal depression. Development of the 10-item Edinburgh postnatal depression scale. Br J Psychiatry. 1987;150:782–6. 3651732 10.1192/bjp.150.6.782

[41] Clark SL, Muthén B. Relating latent class analysis results to variables not included in the analysis. 2009;

[42] Heron J, Croudace TJ, Barker ED, Tilling K. A comparison of approaches for assessing covariate effects in latent class analysis. Longitud Lifecourse Stud. 2015;6(4):420–34.

[43] Vermunt JK. Latent class modeling with covariates: two improved three-step approaches. Polit Anal. 2010;18(4):450–69.

[44] Asparouhov T, Muth B, Muthen B. Auxiliary variables in mixture modeling: 3-step approaches using Mplus. 2013 p. 1–48. https://statmodel.com/examples/webnotes/AuxMixture_submitted_corrected_webnote

[45] Feingold A, Tiberio SS, Capaldi DM. New approaches for examining associations with latent categorical variables: applications to substance abuse and aggression. Psychol Addict Behav. 2014;28(1):257–67. 10.1037/a0031487 23772759 PMC3823694

[46] Sterne JAC, White IR, Carlin JB, Spratt M, Royston P, Kenward MG, et al Multiple imputation for missing data in epidemiological and clinical research: potential and pitfalls. BMJ Br Med J. 2009 29;338:b2393. 19564179 10.1136/bmj.b2393 PMC2714692

[47] Seaman SR, White IR. Review of inverse probability weighting for dealing with missing data. Stat Methods Med Res. 2013;22(3):278–95. 10.1177/0962280210395740 21220355

[48] Muthén LK, Muthén BO. User’s Guide. 7th ed Muthén LK, Muthén BO, editors. Los Angeles, CA: Muthén & Muthén; 2016.

[49] Wolke D, Waylen A, Samara M, Steer C, Goodman R, Ford T, et al Selective drop-out in longitudinal studies and non-biased prediction of behaviour disorders. Br J Psychiatry. 2009;195(3):249–56. 10.1192/bjp.bp.108.053751 19721116 PMC2802508

[50] Warnick EM, Bracken MB, Kasl S. Screening efficiency of the child behavior checklist and strengths and difficulties questionnaire: a systematic review. Child Adolesc Ment Health. 2008;13(3):140–7. 10.1111/j.1475-3588.2007.00461.x 32847173

[51] Hussong AM, Cai L, Curran PJ, Flora DB, Chassin LA, Zucker RA. Disaggregating the distal, proximal, and time-varying effects of parent alcoholism on children’s internalizing symptoms. J Abnorm Child Psychol. 2008;36(3):335–46. 10.1007/s10802-007-9181-9 17891557 PMC2785434

[52] Hussong AM, Huang W, Curran P, Chassin L, Zucker R. Parent alcoholism impacts the severity and timing of children’s externalizing symptoms. J Abnorm Child Psychol. 2010;38(3):367–80. 10.1007/s10802-009-9374-5 20084453 PMC3215275

[53] Chassin L, Pitts SC, DeLucia C, Todd M. A longitudinal study of children of alcoholics: predicting young adult substance use disorders, anxiety, and depression. J Abnorm Psychol. 1999;108(1):106–19. 10066997 10.1037//0021-843x.108.1.106

[54] Christoffersen MN, Soothill K. The long-term consequences of parental alcohol abuse: a cohort study of children in Denmark. J Subst Abuse Treat. 2003;25(2):107–16. 14629993 10.1016/s0740-5472(03)00116-8

[55] Hussong AM, Curran PJ, & Chassin L. Pathways of risk for accelerated heavy alcohol use among adolescent children of alcoholic parents. J Abnorm Child Psychol. 1998;26(6):453–66. 9915652 10.1023/a:1022699701996

[56] Malone SM, McGue M, Iacono WG. Mothers’ maximum drinks ever consumed in 24 hours predicts mental health problems in adolescent offspring. J Child Psychol Psychiatry Allied Discip. 2010;51(9):1067–75. 10.1111/j.1469-7610.2010.02219.x PMC2888884 20085606

[57] Larkby CA, Goldschmidt L, Hanusa BH, & Day NL. Prenatal alcohol exposure is associated with conduct disorder in adolescence: findings from a birth cohort. J Am Acad Child Adolesc Psychiatry. 2012;50(3):1–16. 10.1016/j.jaac.2010.12.004 PMC3042714 21334566

[58] O’Leary CM, Nassar N, Kurinczuk JJ, de Klerk N, Geelhoed E, Elliott EJ, et al Prenatal alcohol exposure and risk of birth defects. Pediatrics. 2010;126(4):e843–50. 10.1542/peds.2010-0256 20876169

[59] Lewis SJ, Zuccolo L, Davey Smith G, Macleod J, Rodriguez S, Draper ES, et al Fetal alcohol exposure and iq at age 8: evidence from a population-based birth-cohort study. PLoS One. 2012;7(11):e49407 10.1371/journal.pone.0049407 23166662 PMC3498109

