# Supplementary material for: Parental alcohol use and risk of behavioral and emotional problems in offspring
Source: PLoS One. 2017 Jun 6;12(6):e0178862. doi: 10.1371/journal.pone.0178862 (PMC5460848; doi:10.1371/journal.pone.0178862)
Supplement: S1 Fig — (A) Flow chart for data availability. Flowchart showing available data for trajectories of childhood conduct problems and partner alcohol consumption at age 4 years. (B). Flow chart for data availability. Flowchart showing available data for adolescent depressive symptoms and partner alcohol consumption at age 4 years. (C). Flow chart for data availability. Flowchart showing available data for adolescent depressive symptoms and partner alcohol consumption at age 12 years. (PDF) [file pone.0178862.s001.pdf]

Online Supplementary Figures

Flow chart for data availability

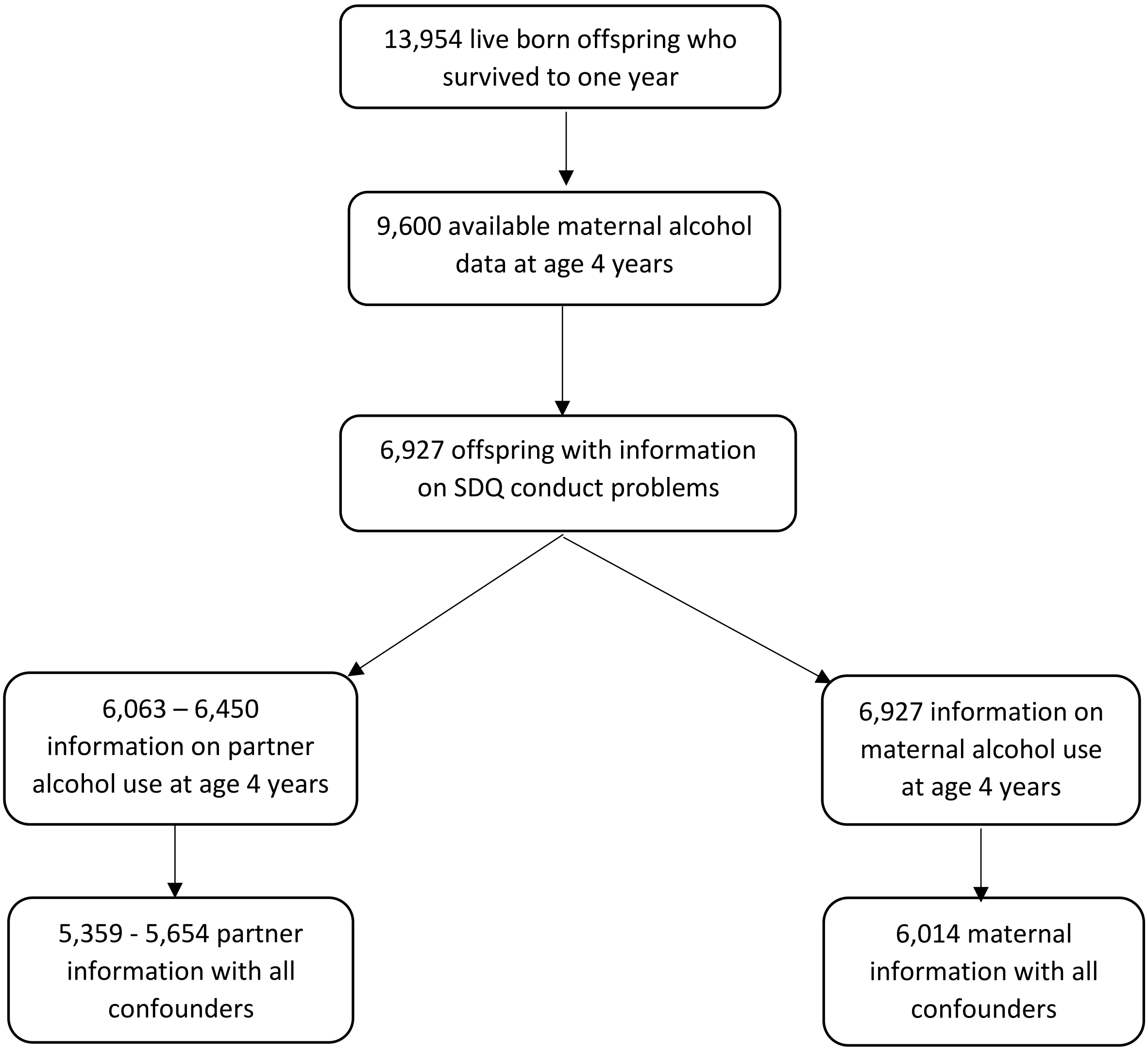

Figure S1a. Flowchart showing available data for trajectories of childhood conduct problems and partner alcohol consumption at age 4 years

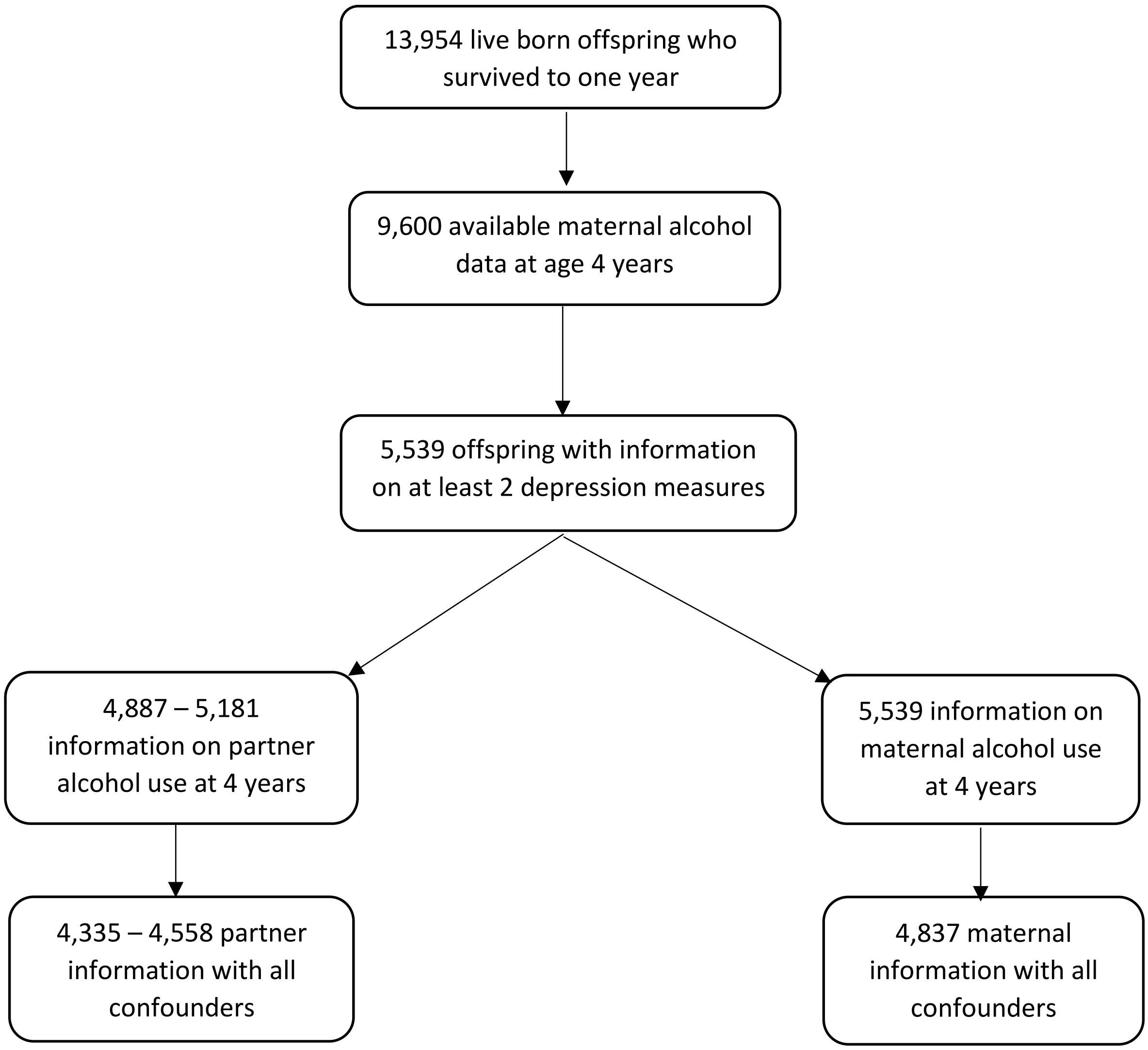

Figure S1b. Flowchart showing available data for adolescent depressive symptoms and partner alcohol consumption at age 4 years

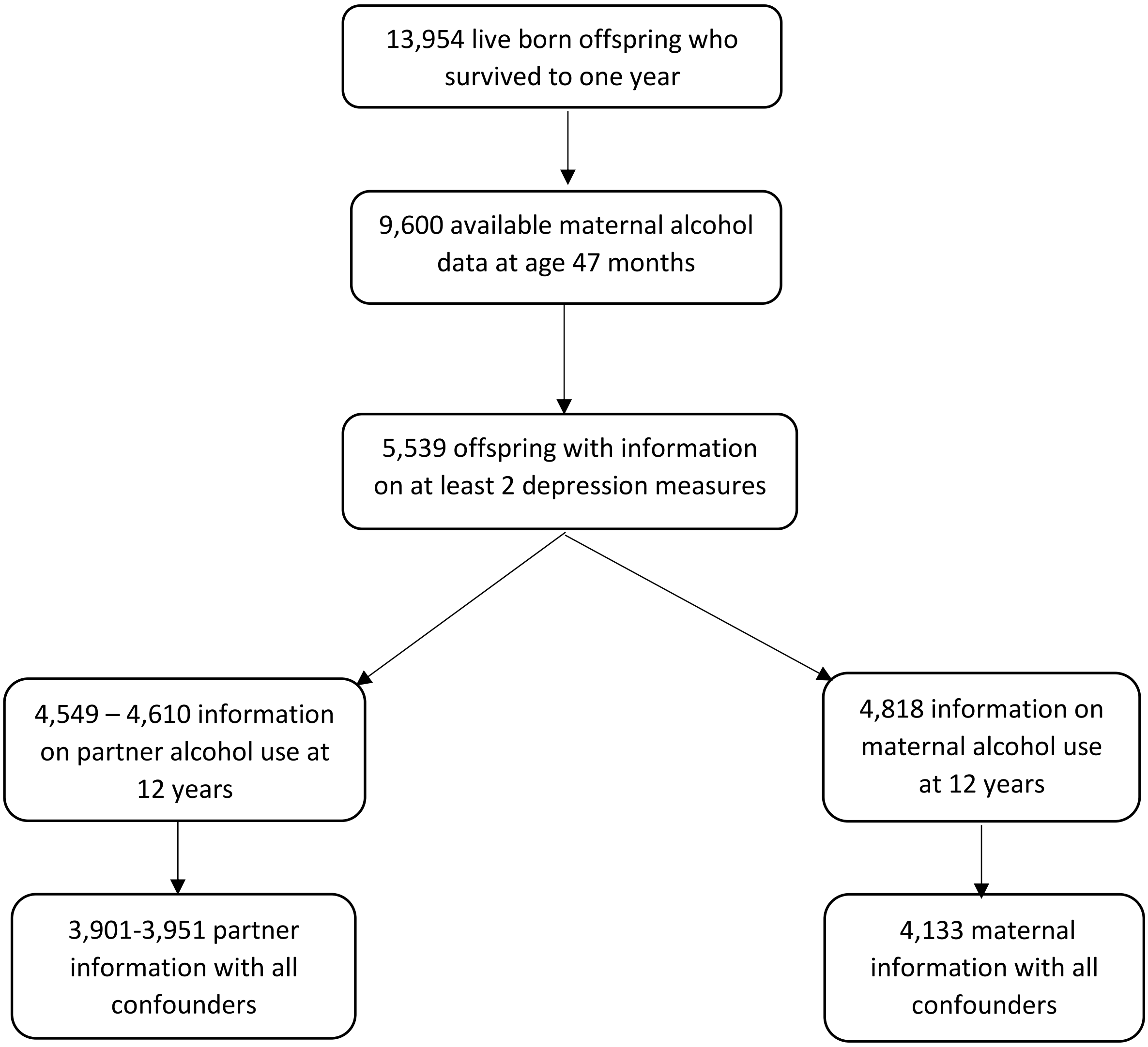

Figure S1c. Flowchart showing available data for adolescent depressive symptoms and partner alcohol consumption at age 12 years
