# Supplementary material for: Parental alcohol use and risk of behavioral and emotional problems in offspring
Source: PLoS One. 2017 Jun 6;12(6):e0178862. doi: 10.1371/journal.pone.0178862 (PMC5460848; doi:10.1371/journal.pone.0178862)
Supplement: S2 Fig — (PDF) [file pone.0178862.s002.pdf]

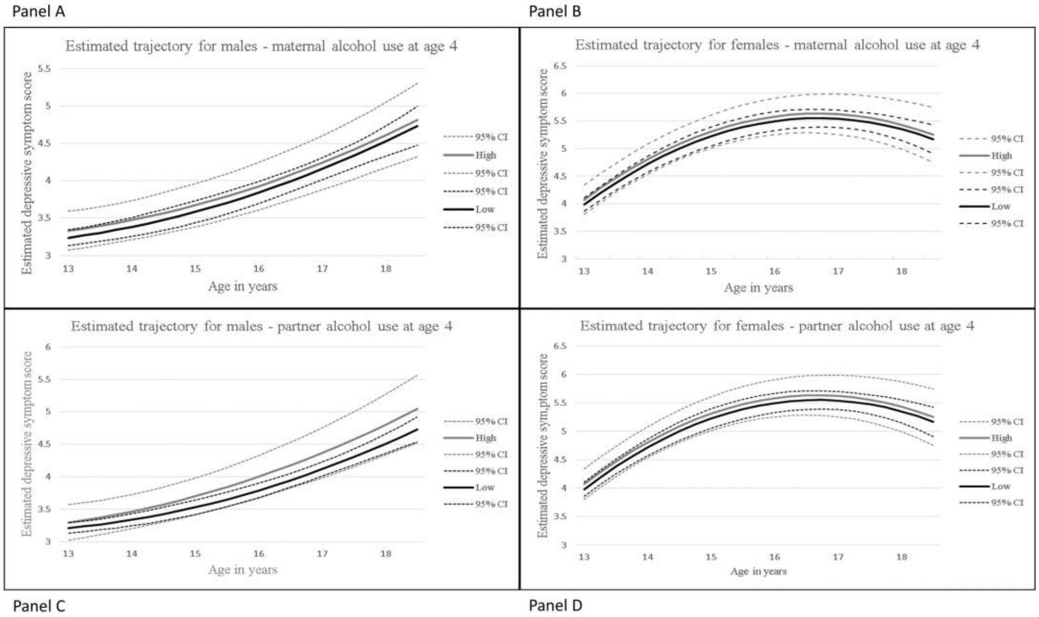

Figure S2. Estimated trajectories of depressive symptoms across adolescence, grouped by heavy and non-heavy parental alcohol use at age 4 years, for maternal alcohol use for males (panel A) and females (panel B), and for partner alcohol use for males (panel C) and females (panel D), with at least two waves of data.
