# Supplementary material for: Parental alcohol use and risk of behavioral and emotional problems in offspring
Source: PLoS One. 2017 Jun 6;12(6):e0178862. doi: 10.1371/journal.pone.0178862 (PMC5460848; doi:10.1371/journal.pone.0178862)
Supplement: S1 Table — (DOCX) [file pone.0178862.s003.docx]

*Table S1.* Descriptive data for key sociodemographic variables – conduct problems

| Exposure | Low conduct group (*n*=5,056) | | Childhood limited (*n*=874) | | Adolescent onset (*n*=616) | | Early-onset persistent (*n*=664) | | *χ², p*-value |
| --- | --- | --- | --- | --- | --- | --- | --- | --- | --- |
| Male gender, *n* (%) | 2,474 | (48.9) | 473 | (54.1) | 306 | (49.7) | 377 | (56.8) | 20.1, <.001 |
| Housing tenure |  |  |  |  |  |  |  |  |  |
| Subsidised rent | 358 | (7.2) | 76 | (8.9) | 59 | (9.8) | 66 | (10.3) |  |
| Private rent | 354 | (7.1) | 96 | (11.3) | 57 | (9.5) | 110 | (17.1) |  |
| Mortgaged | 4,249 | (85.7) | 679 | (79.8) | 484 | (80.7) | 467 | (72.6) | 98.0, <.001 |
| Income, *n* (%) |  |  |  |  |  |  |  |  |  |
| Lowest 20% | 648 | (13.6) | 155 | (19.0) | 104 | (18.1) | 152 | (24.5) |  |
| 2 | 832 | (17.5) | 163 | (20.0) | 121 | (21.0) | 150 | (24.2) |  |
| 3 | 1,006 | (21.2) | 164 | (20.1) | 106 | (18.4) | 105 | (16.9) |  |
| 4 | 1,129 | (23.7) | 157 | (19.2) | 119 | (20.7) | 99 | (16.0) |  |
| Highest 20% | 1,142 | (24.0) | 177 | (21.7) | 126 | (21.9) | 114 | (18.4) | 102.3, <.001 |
| Social economic position, *n* (%) |  |  |  |  |  |  |  |  |  |
| Unskilled or semi-skilled | 164 | (3.4) | 41 | (5.1) | 23 | (4.0) | 39 | (6.5) |  |
| Skilled manual or non-manual | 1,611 | (33.8) | 292 | (36.1) | 197 | (34.4) | 237 | (39.4) |  |
| Managerial and technical | 2,179 | (45.7) | 348 | (43.0) | 264 | (46.1) | 247 | (41.1) |  |
| Professional | 818 | (17.1) | 129 | (15.9) | 89 | (15.5) | 78 | (13.0) | 30.1, <.001 |
| Maternal education, *n* (%) |  |  |  |  |  |  |  |  |  |
| <O level | 2,249 | (45.3) | 333 | (39.3) | 256 | (42.7) | 230 | (35.8) |  |
| O level | 1,762 | (35.5) | 303 | (35.7) | 213 | (35.6) | 225 | (34.5) |  |
| >O level | 958 | (19.3) | 212 | (25.0) | 130 | (21.7) | 188 | (29.2) | 50.3, <.001 |
| Parity, *n* (%) |  |  |  |  |  |  |  |  |  |
| First | 2,319 | (47.0) | 376 | (44.5) | 302 | (50.4) | 294 | (45.4) |  |
| Second | 1,775 | (35.9) | 310 | (36.7) | 207 | (34.6) | 217 | (33.5) |  |
| Third+ | 845 | (17.1) | 159 | (18.8) | 90 | (15.0) | 136 | (21.0) | 12.2, .057 |
| Smoking in pregnancy, *n* (%) |  |  |  |  |  |  |  |  |  |
| No | 4,161 | (82.3) | 670 | (76.7) | 473 | (47.3) | 458 | (69.0) |  |
| Yes | 895 | (17.7) | 204 | (23.3) | 143 | (23.2) | 206 | (31.0) | 76.9, <.001 |
|  |  |  |  |  |  |  |  |  |  |
| Maternal age, Mean (SD) | 29.4 | (4.4) | 29.0 | (4.8) | 28.6 | (4.6) | 28.3 | (4.8) |  |
|  |  |  |  |  |  |  |  |  |  |
| Maternal depression, Mean (SD) | 6.1 | (4.6) | 7.2 | (5.1) | 8.0 | (5.1) | 8.6 | (5.2) |  |
|  |  |  |  |  |  |  |  |  |  |
