# Supplementary material for: Parental alcohol use and risk of behavioral and emotional problems in offspring
Source: PLoS One. 2017 Jun 6;12(6):e0178862. doi: 10.1371/journal.pone.0178862 (PMC5460848; doi:10.1371/journal.pone.0178862)
Supplement: S2 Table — (DOCX) [file pone.0178862.s004.docx]

*Table S2.* Descriptive data for key sociodemographic variables – depressive symptoms

|  |  |  |  |  |  |  |  |
| --- | --- | --- | --- | --- | --- | --- | --- |
| Exposure | *N* | No SMFQ | 1 measure | 2 measures | 3 measures | 4 measures | *χ², p*-value |
| Male | 7,205 | 3,463 (56.8) | 805 (47.9) | 1,015 (56.8) | 868 (47.3) | 1,054 (41.3) | 217.6, <.001 |
| Housing tenure |  |  |  |  |  |  |  |
| Subsidised rent | 1,385 | 744 (13.7) | 185 (11.6) | 144 (8.4) | 145 (8.2) | 167 (6.7) |  |
| Private rent | 2,080 | 1,361 (25.13) | 234 (14.7) | 201 (11.7) | 155 (8.7) | 129 (5.2) |  |
| Mortgaged | 9,540 | 3,311 (61.1) | 1,172 (73.7) | 1,376 (80.0) | 1,476 (83.1) | 2,205 (88.2) | 870.6, <.001 |
| Income |  |  |  |  |  |  |  |
| Lowest 20% | 1,989 | 888 (28.8) | 297 (22.5) | 296 (19.6) | 245 (15.1) | 263 (11.0) |  |
| 2 | 1,966 | 661 (21.5) | 270 (20.4) | 307 (20.3) | 325 (20.0) | 403 (16.9) |  |
| 3 | 1,976 | 566 (18.4) | 272 (20.6) | 338 (22.3) | 323 (19.9) | 477 (20.0) |  |
| 4 | 1,985 | 499 (16.2) | 251 (19.0) | 290 (19.2) | 361 (22.3) | 584 (24.5) |  |
| Highest 20% | 2,007 | 465 (15.1) | 233 (17.6) | 282 (18.6) | 368 (22.7) | 659 (27.6) | 366.2, <.001 |
| Social economic position |  |  |  |  |  |  |  |
| Unskilled or semi-skilled | 682 | 366 (8.5) | 84 (5.9) | 94 (5.9) | 71 (4.2) | 67 (2.8) |  |
| Skilled manual or non-manual | 4,476 | 1,954 (45.1) | 591 (41.8) | 617 (38.8) | 625 (36.7) | 689 (28.3) |  |
| Managerial and technical | 4,799 | 1,628 (37.6) | 557 (39.4) | 706 (44.4) | 753 (44.2) | 1,155 (47.4) |  |
| Professional | 1,524 | 384 (8.9) | 182 (12.9) | 174 (10.9) | 256 (15.0) | 528 (21.7) | 462.0, <.001 |
| Maternal education |  |  |  |  |  |  |  |
| <O level | 4,385 | 1,207 (24.8) | 498 (32.6) | 575 (34.1) | 762 (42.8) | 1,343 (53.3) |  |
| O level | 4,287 | 1,656 (34.0) | 523 (34.2) | 644 (38.2) | 647 (36.3) | 817 (32.4) |  |
| >O level | 3,723 | 2,014 (41.3) | 509 (33.3) | 467 (27.7) | 373 (20.9) | 360 (14.3) | 900.1, <.001 |
| Parity |  |  |  |  |  |  |  |
| First | 5,765 | 2,224 (41.8) | 664 (41.7) | 742 (43.1) | 868 (49.0) | 1,267 (50.7) |  |
| Second | 4,531 | 1,846 (34.7) | 579 (36.3) | 638 (37.1) | 613 (34.6) | 855 (34.2) |  |
| Third | 2,612 | 1,255 (23.6) | 351 (22.0) | 341 (19.8) | 289 (16.3) | 376 (15.1) | 124.7, <.001 |
| Smoking in pregnancy |  |  |  |  |  |  |  |
| No | 9,672 | 3,543 (58.1) | 1,153 (68.6) | 1,336 (74.8) | 1,487 (81.1) | 2,153 (84.3) |  |
| Yes | 4,282 | 2,554 (41.9) | 528 (31.4) | 451 (25.2) | 347 (18.9) | 402 (15.7) | 773.1, <.001 |
|  |  |  |  |  |  |  |  |
| Maternal age, Mean (SD) | 13, 954 | 6,097 (43.7) | 1,681 (12.0) | 1,787 (12.8) | 1,834 (13.1) | 2,555 (18.3) |  |
|  |  |  |  |  |  |  |  |
| Maternal depression, Mean (SD) | 12,014 | 4,718 (39.2) | 1,470 (12.2) | 1,641 (13.7) | 1,737 (14.5) | 2,448 (20.4) |  |
