# Supplementary material for: Parental alcohol use and risk of behavioral and emotional problems in offspring
Source: PLoS One. 2017 Jun 6;12(6):e0178862. doi: 10.1371/journal.pone.0178862 (PMC5460848; doi:10.1371/journal.pone.0178862)
Supplement: S3 Table — Note: EOP: early onset persistent, CL: childhood limited, AO: Adolescent onset, the Low group was used as the reference group; SEP: social economic position was grouped into 4 categories: 1: unskilled or semiskilled manual; 2: skilled manual or nonmanual; 3: managerial and technical; and 4: professional. (DOCX) [file pone.0178862.s005.docx]

*Outcome #1:* Group-based trajectories of childhood conduct problems

Female gender, living in rented accommodation, maternal smoking in the antenatal period, and low parental income were associated with baseline and growth of conduct problem across all groups compared to offspring who were in the ‘low’ conduct problems group. Having three or more children in the family, low maternal education were associated with EOP and CL conduct trajectory groups. Medium social status was associated with lower EOP and CL conduct trajectory groups. Associations between each of the background socioeconomic variables and trajectory classes were examined using the Wald χ² test (S3Table).

*Table S3.* Univariable associations between demographic variables and trajectories of conduct problems

|  |  | EOP | CL | AO |  |
| --- | --- | --- | --- | --- | --- |
|  | *n* (%) | OR(95% CI) | OR(95% CI) | OR(95% CI) | *test* *p* |
| **Gender** |  |  |  |  |  |
| Females | 3,428 (49.5) | 0.73 (.62, .86) | 0.79 (0.68, 0.91) | 0.98 (0.83, 1.17) |  |
| Males | 3,499 (50.5) | ref |  |  | <.001 |
| **Tenure** |  |  |  |  |  |
| Subsidised rent | 523 (7.7) | 1.73 (1.30, 2.29) | 1.34 (1.02, 1.75) | 1.39 (1.02, 1.88) |  |
| Private rent | 592 (8.7) | 2.73 (2.15, 3.47) | 1.74 (1.37, 2.22) | 1.39 (1.03, 1.88) |  |
| Mortgaged | 5,673 (83.6) | ref |  |  | <.001 |
| **Parity** |  |  |  |  |  |
| Third+ | 1,184 (17.5) | 1.27 (1.02, 1.59) | 1.17 (0.95, 1.43) | 0.82 (0.64, 1.06) |  |
| Second | 2,401 (35.5) | 0.95 (0.78, 1.15) | 1.10 (0.93, 1.29) | 0.91 (0.75, 1.10) |  |
| First | 3,178 (47.0) | ref |  |  | .05 |
| **Mat education** |  |  |  |  |  |
| <O level | 2,970 (43.7) | 1.25 (1.03, 1.53) | 1.17 (0.99, 1.39) | 1.08 (0.89, 1.31) |  |
| O level | 2,404 (35.4) | 1.90 (1.54, 2.35) | 1.50 (1.23, 1.81) | 1.24 (0.99, 1.56) |  |
| >O level | 1,424 (21.0) | ref |  |  | <.001 |
| **Mat smoking** |  |  |  |  |  |
| Yes | 909 (16.4) | 2.45 (1.99, 3.01) | 1.57 (1.28, 1.93) | 1.47 (1.15, 1.87) |  |
| No | 5,553 (83.4) | ref |  |  | <.001 |
| **Income** |  |  |  |  |  |
| Low 20% | 1,027 (15.5) | 2.31 (1.77, 3.00) | 1.59 (1.25, 2.02) | 1.45 (1.10, 1.92) |  |
| 40% | 1,248 (18.9) | 1.80 (1.39, 2.36) | 1.28 (1.01, 1.61) | 1.30 (0.99, 1.70) |  |
| 60% | 1,349 (20.4) | 1.01 (0.76, 1.34) | 1.03 (0.81, 1.30) | 0.95 (0.72, 1.25) |  |
| 80% | 1,469 (22.2) | 0.88 (0.66, 1.17) | 0.90 (0.71, 1.13) | 0.94 (0.72, 1.23) |  |
| High 100% | 1,528 (23.1) | ref |  |  | <.001 |
| **SEP** |  |  |  |  |  |
| 1 | 251 (3.9) | 0.68 (0.46, 1.01) | 0.73 (0.50, 1.06) | 0.88 (0.55, 1.41) |  |
| 2 | 2,250 (34.6) | 0.52 (0.35, .78) | 0.63 (0.44, .91) | 0.84 (0.53, 1.33) |  |
| 3 | 2,929 (45.0) | 0.44 (0.29, .69) | 0.63 (0.42, .93) | 0.77 (0.47, 1.27) |  |
| 4 | 1,080 (16.6) | ref |  |  | <.001 |
| **Mat depression** |  |  |  |  |  |
| Linear term | 6,612 | 1.11 (1.09, 1.13) | 1.06 (1.04, 1.07) | 1.08 (1.06, 1.10) | <.001 |
|  |  |  |  |  |  |

*Note: EOP: early onset persistent, CL: childhood limited, AO: Adolescent onset, the Low group was used as the reference group; SEP: social economic position was grouped into 4 categories: 1: unskilled or semiskilled manual; 2: skilled manual or nonmanual; 3: managerial and technical; and 4: professional*
