# Supplementary material for: Parental alcohol use and risk of behavioral and emotional problems in offspring
Source: PLoS One. 2017 Jun 6;12(6):e0178862. doi: 10.1371/journal.pone.0178862 (PMC5460848; doi:10.1371/journal.pone.0178862)
Supplement: S4 Table — Note: SEP: social economic position was grouped into 4 categories: 1: unskilled or semiskilled manual; 2: skilled manual or nonmanual; 3: managerial and technical; and 4: professional. (DOCX) [file pone.0178862.s006.docx]

*Outcome #2:* Offspring depressive symptoms

Maternal smoking, female gender, living in rented accommodation, and maternal depression in the antenatal period were associated with baseline and growth of depressive symptoms. Offspring whose mothers had lower levels of education showed increased growth of depressive symptoms across adolescence. Low parental income and social status were associated with growth of depressive symptoms across adolescence. Associations between each of the background socioeconomic variables and baseline and growth parameters were examined using the Wald χ² test (S4 Table).

*Table S4.* Univariable associations between demographic variables and offspring depressive symptoms

|  | Depressive symptoms | | | | |
| --- | --- | --- | --- | --- | --- |
|  | *n* | Intercept | *test p* | Slope | *test p* |
|  |  | b (95% CI) |  | b (95% CI) |  |
| **Gender** |  |  |  |  |  |
| Females | 2,937 | 0.28 (.23, .34) | .000 | 0.24 (.15, .33) |  |
| Males | 3,239 | ref |  |  | <.001 |
| **Tenure** |  |  |  |  |  |
| Subsidised rent | 456 | 0.15 (.04, .26) |  | -0.00 (-.19, .18) |  |
| Private rent | 485 | 0.15 (.03, .26) | .945 | 0.24 (.06, .43) |  |
| Mortgage | 5,057 | ref |  |  | .06 |
| **Parity** |  |  |  |  |  |
| Third+ | 1,006 | 0.08 (-.00, .16) | .101 | 0.10 (-.02, .23) |  |
| Second | 2,106 | 0.01 (-.05, .07) |  | 0.03 (-.07, .12) |  |
| First | 2,877 | ref |  |  | .27 |
| **Mat education** |  |  |  |  |  |
| <O level | 2,680 | 0.00 (-.07, .07) |  | 0.16 (.07, .26) |  |
| O level | 2,108 | -0.01 (-.08, .07) | .899 | 0.27 (.15, .40) |  |
| >O level | 1,200 | ref |  |  | .09 |
| **Mat smoking** |  |  |  |  |  |
| Yes | 1,200 | 0.25 (.15, .34) | .000 | 0.21 (.06, .37) |  |
| No | 4,976 | ref |  |  | .01 |
| **Income** |  |  |  |  |  |
| Low 20% | 804 | 0.10 (-.00, .20) |  | 0.22 (.07, .37) |  |
| 40% | 1,035 | 0.11 (.02, .20) |  | 0.15 (.01, .28) |  |
| 60% | 1,138 | 0.02 (-.06, .11) |  | 0.14 (.10, .26) |  |
| 80% | 1,235 | -0.00 (-.09, .08) | .054 | 0.04 (-.08, .16) |  |
| High 100% | 1,309 | ref |  |  | .11 |
| **SEP** |  |  |  |  |  |
| 1 | 232 | -0.10 (-.24, .04) |  | -0.28 (-.57, .01) |  |
| 2 | 1,931 | -0.08 (-.21, .06) |  | -0.38 (-.67, -.10) |  |
| 3 | 2,614 | -0.11 (-.26, .03) | .676 | -0.52 (-.81, -.22) |  |
| 4 | 958 | ref |  |  | <.001 |
| **Mat depression** |  |  |  |  |  |
| Linear term | 5,826 | 0.13 (.10, .16) | .000 | 0.08 (.03, .13) | <.001 |

*Note: SEP: social economic position was grouped into 4 categories: 1: unskilled or semiskilled manual; 2: skilled manual or nonmanual; 3: managerial and technical; and 4: professional*
