# Supplementary material for: Parental alcohol use and risk of behavioral and emotional problems in offspring
Source: PLoS One. 2017 Jun 6;12(6):e0178862. doi: 10.1371/journal.pone.0178862 (PMC5460848; doi:10.1371/journal.pone.0178862)
Supplement: S5 Table — (DOCX) [file pone.0178862.s007.docx]

*Table S5.* Selective attrition for childhood conduct problems and adolescent depressive symptoms

|  | Conduct problems | | | Depressive symptoms | | |
| --- | --- | --- | --- | --- | --- | --- |
|  |  |  |  |  |  |  |
|  | Not available | Available |  | Not available | Available |  |
| Sex | *n* (%) | *n* (%) | OR (95% CI) | *n* (%) | *n* (%) | OR (95% CI) |
| Male | 1,466 (54.8) | 3,499 (50.5) | 0.84 (.77, .92) | 2,304 (56.7) | 2,661 (48.0) | 0.71 (.65, .76) |
| Female | 1,207 (45.2) | 3,428 (49.5) | ref | 1,757 (43.3) | 2,878 (52.0) | ref |
| Intentional pregnancy |  |  |  |  |  |  |
| No | 890 (34.8) | 1,729 (25.4) | ref | 1,272 (32.5) | 1,347 (24.7) | ref |
| Yes | 1,666 (65.2) | 5,089 (74.6) | 1.57 (1.42, 1.73) | 2,644 (67.5) | 4,111 (75.3) | 1.47 (1.34, 1.61) |
| Mould on walls |  |  |  |  |  |  |
| No | 1,398 (54.9) | 3,457 (51.0) | ref | 1,820 (46.6) | 2,765 (51.1) | ref |
| Yes | 1,149 (45.1) | 3,322 (49.0) | 1.16 (1.06, 1.28) | 2,090 (53.5) | 2,651 (49.0) | 1.10 (1.01, 1.20) |
| Marital status |  |  |  |  |  |  |
| Single | 595 (23.2) | 885 (13.0) | ref | 2,594 (66.1) | 4,165 (76.2) | ref |
| First | 1,600 (62.5) | 5,159 (75.5) | 0.46 (.40, .52) | 778 (19.8) | 702 (12.8) | 0.56 (.50, .63) |
| Marriage 2 or 3 | 173 (6.8) | 334 (4.9) | 0.77 (.62, .95) | 252 (6.4) | 255 (4.7) | 0.89 (.73, 1.09) |
| Widowed/divorced/separated | 192 (7.5) | 453 (6.6) | 0.63 (.52, .77) | 298 (7.6) | 347 (6.3) | 0.77 (.64, .93) |
| Car ownership |  |  |  |  |  |  |
| No | 329 (12.9) | 363 (5.4) | 2.62 (2.23, 3.06) | 449 (11.5) | 243 (4.5) | 2.75 (2.34, 3.23) |
| Yes | 2,224 (87.1) | 6,423 (94.7) | ref | 3,472 (88.6) | 5,122 (93.7) | ref |
| History of depression |  |  |  |  |  |  |
| No | 2,209 (88.9) | 6,338 (93.1) | ref | 3,474 (90.1) | 5,073 (93.3) | ref |
| Yes | 277 (11.1) | 469 (6.9) | 1.69 (1.44, 1.98) | 383 (9.9) | 363 (6.7) | 1.54 (1.33, 1.79) |
| Smoking |  |  |  |  |  |  |
| No | 1,752 (65.5) | 5,553 (80.2) | ref | 2,769 (68.2) | 4,536 (81.9) | ref |
| Yes | 921 (34.5) | 1,374 (19.8) | 2.12 (1.92, 2.34) | 1,292 (31.8) | 1,003 (18.1) | 2.11 (1.91, 2.32) |
| Maternal age | Mean (SD) | Mean (SD) |  | Mean (SD) | Mean (SD) |  |
|  | 27.3 (4.8) | 29.2 (4.5) | 0.92 (.91, .93) | 27.8 (4.9) | 29.3 (4.5) | 0.93 (.93, .94) |
|  |  |  |  |  |  |  |
