# Supplementary material for: Parental alcohol use and risk of behavioral and emotional problems in offspring
Source: PLoS One. 2017 Jun 6;12(6):e0178862. doi: 10.1371/journal.pone.0178862 (PMC5460848; doi:10.1371/journal.pone.0178862)
Supplement: S6 Table — (A) Childhood conduct problem trajectories and parental alcohol consumption–unweighted estimates (low group–reference group). Note: 1Maternal reports of partner’s alcohol consumption; 2Univariable multinomial logistic regression models; 3Multinomial logistic regression models adjusted for maternal age at delivery, parity, Social economic position, maternal education, maternal smoking during first trimester in pregnancy, housing tenure, income, and maternal depressive symptoms at 32 weeks gestation; CL: childhood limited, AO: adolescent onset, EOP: early onset persistent, the Low conduct problems class was used as the reference group. (B). Heavy parental alcohol consumption (assessed at age 4 years using binary alcohol measures) and childhood conduct problem trajectories–unweighted estimates. Note: 1Maternal reports of partner’s alcohol consumption; CL: childhood limited, AO: adolescent onset, EOP: early onset persistent, the Low conduct problems class was used as the reference group. 2Models adjusted for maternal age at delivery, parity, social economic position, maternal education, maternal smoking during first trimester in pregnancy, housing tenure, income, and maternal depressive symptoms at 32 weeks gestation. (DOCX) [file pone.0178862.s008.docx]

*Table S6a.* Childhood conduct problem trajectories and parental alcohol consumption – unweighted estimates (low group – reference group)

|  | Model 1 | | | |  | Model 2 | | | |  |
| --- | --- | --- | --- | --- | --- | --- | --- | --- | --- | --- |
|  |  | CL | AO | EOP |  |  | CL | AO | EOP |  |
|  | *n* | OR  (95% CI) | OR  (95% CI) | OR  (95% CI) | *p* | *n* | OR  (95% CI) | OR  (95% CI) | OR  (95% CI) | *p* |
| Maternal alcohol use in units – linear term | 6,927 | 1.00  (.99, 1.01) | 0.99  (.98, 1.01) | 0.98  (.96, 1.00) | .07 | 6,014 | 1.00  (.99, 1.01) | 0.98  (.97, 1.00) | 0.98  (.96, 1.00) | .47 |
| Partner drinking 4+ units^1^ – linear term | 6,063 | 1.01  (.93, 1.09) | 1.05  (.96, 1.17) | 0.98  (.90, 1.06) | .81 | 5,359 | 1.02  (.93, 1.12) | 1.00  (.88, 1.12) | 0.98  (.90, 1.08) | .58 |

*Note: ^1^Maternal reports of partner’s alcohol consumption; ^2^Univariable multinomial logistic regression models; ^3^Multinomial logistic regression models adjusted for maternal age at delivery, parity, Social economic position, maternal education, maternal smoking during first trimester in pregnancy, housing tenure, income, and maternal depressive symptoms at 32 weeks gestation; CL: childhood limited, AO: adolescent onset, EOP: early onset persistent, the Low conduct problems class was used as the reference group*

*Table S6b.* Heavy parental alcohol consumption (assessed at age 4 years using binary alcohol measures) and childhood conduct problem trajectories – unweighted estimates

|  | Model 1 | | | | | Model 2^2^ | | | |  |
| --- | --- | --- | --- | --- | --- | --- | --- | --- | --- | --- |
|  | *N* | OR  (95% CI) | OR  (95% CI) | OR  (95% CI) | *p* | *N* | OR  (95% CI) | OR  (95% CI) | OR  (95% CI) | *p* |
|  |  | CL | AO | EOP |  |  | CL | AO | EOP |  |
| Maternal drinking ≥21 units (8.7%) | 6,927 | 1.50  (1.07, 2.09) | 0.77  (.44, 1.35) | 0.81  (.55, 1.21) | .15 | 6,014 | 1.48  (1.01, 2.15) | 0.61  (.30, 1.24) | 0.76  (.48, 1.20) | .22 |
| Partner drinking 4+ units *everyday^1^* (5.2%) | 6,063 | 0.84  (.43, 1.63) | 1.20  (.59, 2.43) | 1.22  (.77, .1.93) | .31 | 5,359 | 0.90  (.44, 1.82) | 0.84  (.30, 2.38) | 1.15  (.70, 1.90) | .40 |

*Note: ^1^Maternal reports of partner’s alcohol consumption; CL: childhood limited, AO: adolescent onset, EOP: early onset persistent, the Low conduct problems class was used as the reference group.* ^2^*Models adjusted for maternal age at delivery, parity, social economic position, maternal education, maternal smoking during first trimester in pregnancy, housing tenure, income, and maternal depressive symptoms at 32 weeks gestation*
