# Supplementary material for: Parental alcohol use and risk of behavioral and emotional problems in offspring
Source: PLoS One. 2017 Jun 6;12(6):e0178862. doi: 10.1371/journal.pone.0178862 (PMC5460848; doi:10.1371/journal.pone.0178862)
Supplement: S7 Table — (A) Parental alcohol use (assessed at age 4 and 12 years using linear alcohol measures) and adolescent depressive symptoms–unweighted estimates. Note. 1Maternal reports of partner’s alcohol consumption; 2Univariable linear regression models; 3Models adjusted for maternal age at delivery, parity, social economic position, maternal education, maternal smoking during first trimester in pregnancy, housing tenure, income, and maternal depressive symptoms at 32 weeks gestation. (B). Heavy parental alcohol use (assessed at ages 4 and 12 years using binary alcohol measures) and adolescent offspring depressive symptoms–unweighted estimates. Note: 1Maternal reports of partner’s alcohol consumption; 2Univariable linear regression models; 3Models adjusted for maternal age at delivery, parity, social economic position, maternal education, maternal smoking during first trimester in pregnancy, housing tenure, income, and maternal depressive symptoms at 32 weeks gestation. (DOCX) [file pone.0178862.s009.docx]

*Table S7a.* Parental alcohol use (assessed at age 4 and 12 years using linear alcohol measures) and adolescent depressive symptoms – unweighted estimates

|  |  |  | Model 1 | | | |  | Model 2 | | | | | | |
| --- | --- | --- | --- | --- | --- | --- | --- | --- | --- | --- | --- | --- | --- | --- |
|  |  | Intercept | |  | Slope |  | |  | Intercept |  | Slope |  | |  |
|  | *N* | *b* (95% CI) | | *p* | *b* (95% CI) | *p* | | *N* | *b* (95% CI) | *p* | *b* (95% CI) | *p* | |  |
| **Age 4 years** |  |  | |  |  |  | |  |  |  |  |  | |  |
| Maternal alcohol use in units - linear term | 5,539 | .003 (-.01, .01) | | .52 | -.009 (-.03, .02) | .45 | | 4,837 | .001 (-.01, .01) | .90 | .003 (-.02, .03) | | .84 |  |
| Partner drinking 4+ units^1^ – linear term | 4,887 | -.045 (-.10, .01) | | .13 | .046 (-.13, .22) | .60 | | 4,335 | -.068 (-.13, -.01) | .03 | .077 (-.11, .26) | | .41 |  |
| **Age 12 years** |  |  | |  |  |  | |  |  |  |  | |  |  |
| Maternal alcohol use in units - linear term | 4,818 | .005 (-.00, .01) | | .18 | .013 (-.01, .04) | .24 | | 4,133 | .002 (-.01, .01) | .64 | .022 (-.00, .05) | | .06 |  |
| Partner drinking 4+ units – linear term^1^ | 4,549 | -.012 (-.07, .04) | | .66 | .028 (-.14, .19) | .74 | | 3,901 | .026 (-.03, .09) | .40 | .048 (-.13, .22) | | .59 |  |

*Note. ^1^Maternal reports of partner’s alcohol consumption; ^2^Univariable linear regression models;* ^3^*Models adjusted for maternal age at delivery, parity, social economic position, maternal education, maternal smoking during first trimester in pregnancy, housing tenure, income, and maternal depressive symptoms at 32 weeks gestation*

*Table S7b.* Heavy parental alcohol use (assessed at ages 4 and 12 years using binary alcohol measures) and adolescent offspring depressive symptoms – unweighted estimates

|  |  |  | Model 1 | | | |  | Model 2 | | | | | | |
| --- | --- | --- | --- | --- | --- | --- | --- | --- | --- | --- | --- | --- | --- | --- |
|  |  | Intercept | |  | Slope |  | |  | Intercept |  | Slope |  | |  |
|  | *N* | *b* (95% CI) | | *p* | *b* (95% CI) | *p* | | *N* | *b* (95% CI) | *p* | *b* (95% CI) | *p* | |  |
| **Age 4 years** |  |  | |  |  |  | |  |  |  |  |  | |  |
| Maternal drinking ≥21 units (9.1%) | 5,539 | .084 (-.18, .34) | | .53 | .046 (-.76, .85) | .91 | | 4,837 | .024 (-.25, .30) | .86 | .264 (-.58, 1.11) | | .54 |  |
| Partner drinking 4+ units everyday^1^ (5.2%) | 4,887 | .046 (-.33, .42) | | .81 | .569 (-.72, 1.86) | .39 | | 4,335 | -.067 (-.48, .34) | .75 | 1.06 (-.32, 2.44) | | .13 |  |
| **Age 12 years** |  |  | |  |  |  | |  |  |  |  | |  |  |
| Maternal drinking ≥21 units (14.9%) | 4,818 | .004 (-.18, .27) | | .70 | .532 (-.17, 1.23) | .14 | | 4,133 | -.004 (-.24, .24) | .97 | . (-.11, 1.38) | | .09 |  |
| Partner drinking 4+ units everyday^1^ (7.4%) | 4,549 | .392 (.03, .76) | | .04 | .52 (-.47, 1.52) | .30 | | 3,901 | .004 (-.37, .38) | .98 | .801 (-.22, 1.82) | | .13 |  |

*Note: ^1^Maternal reports of partner’s alcohol consumption; ^2^Univariable linear regression models;* ^3^*Models adjusted for maternal age at delivery, parity, social economic position, maternal education, maternal smoking during first trimester in pregnancy, housing tenure, income, and maternal depressive symptoms at 32 weeks gestation*
