# Supplementary material for: Parental alcohol use and risk of behavioral and emotional problems in offspring
Source: PLoS One. 2017 Jun 6;12(6):e0178862. doi: 10.1371/journal.pone.0178862 (PMC5460848; doi:10.1371/journal.pone.0178862)
Supplement: S8 Table — (A) Childhood conduct problem trajectories and parental alcohol consumption–unweighted estimates (low group–reference group). Note: 1Maternal reports of partner’s alcohol consumption; Model 1 adjusted for maternal age at delivery, parity, social economic position, maternal education, maternal smoking during first trimester in pregnancy, housing tenure, income, and maternal depressive symptoms at 32 weeks gestation; Model 2 further adjusted for maternal alcohol use at 18 weeks gestation; CL: childhood limited, AO: adolescent onset, EOP: early onset persistent, the Low conduct problems class was used as the reference group. (B). Heavy parental alcohol consumption (assessed at age 4 years using binary alcohol measures) and childhood conduct problem trajectories–unweighted estimates. Note: 1Maternal reports of partner’s alcohol consumption; Model 1 adjusted for maternal age at delivery, parity, social economic position, maternal education, maternal smoking during first trimester in pregnancy, housing tenure, income, and maternal depressive symptoms at 32 weeks gestation; Model 2 further adjusted for maternal alcohol use at 18 weeks gestation; CL: childhood limited, AO: adolescent onset, EOP: early onset persistent, the Low conduct problems class was used as the reference group. (DOCX) [file pone.0178862.s010.docx]

Information on maternal alcohol use during the first trimester of pregnancy was assessed using a questionnaire at 18 weeks gestation. Mothers were asked about the frequency of drinking with response options being: ‘never’, ‘less than 1 glass per week’, ‘at least 1 glass per week’, ‘1-2 glasses a day’, ‘3-9 glasses a day’, or ‘more than 10 glasses a day’. For our analyses, mothers who drank 1 or more glasses a day were combined, resulting in a three-level variable ‘never’: *n* = 4,174 (44.6%), ‘less than 1 glass per week’: *n* = 3728 (53.6%), and ‘at least 1 glass per week’: *n* = 172 (1.8%). One glass was equivalent to one unit (8g) of alcohol.

*Table S8a.* Childhood conduct problem trajectories and parental alcohol consumption – unweighted estimates (low group – reference group)

|  | Model 1 | | | |  | Model 2 | | | |  |
| --- | --- | --- | --- | --- | --- | --- | --- | --- | --- | --- |
|  |  | CL | AO | EOP |  |  | CL | AO | EOP |  |
|  | *N* | OR  (95% CI) | OR  (95% CI) | OR  (95% CI) | *p* | *n* | OR  (95% CI) | OR  (95% CI) | OR  (95% CI) | *p* |
| Maternal alcohol use in units – linear term | 6,014 | 1.00  (.99, 1.01) | 0.98  (.97, 1.00) | 0.98  (.96, 1.00) | .47 | 5,757 | 1.00  (.99, 1.01) | 0.99  (.98, 1.01) | 0.99  (.98, 1.01) | .59 |
| Partner drinking 4+ units^1^ – linear term | 5,359 | 1.02  (.93, 1.12) | 1.00  (.88, 1.12) | 0.98  (.90, 1.08) | .58 | 5,146 | 1.02  (.93, 1.12) | 1.01  (.91, 1.11) | 0.98  (.90, 1.11) | .68 |

*Note: ^1^Maternal reports of partner’s alcohol consumption; Model 1 adjusted for maternal age at delivery, parity, social economic position, maternal education, maternal smoking during first trimester in pregnancy, housing tenure, income, and maternal depressive symptoms at 32 weeks gestation; Model 2 further adjusted for maternal alcohol use at 18 weeks gestation; CL: childhood limited, AO: adolescent onset, EOP: early onset persistent, the Low conduct problems class was used as the reference group.*

*Table S8b.* Heavy parental alcohol consumption (assessed at age 4 years using binary alcohol measures) and childhood conduct problem trajectories – unweighted estimates

|  | Model 1 | | | | | Model 2 | | | |  |
| --- | --- | --- | --- | --- | --- | --- | --- | --- | --- | --- |
|  | *N* | OR  (95% CI) | OR  (95% CI) | OR  (95% CI) | *p* | *N* | OR  (95% CI) | OR  (95% CI) | OR  (95% CI) | *p* |
|  |  | CL | AO | EOP |  |  | CL | AO | EOP |  |
| Maternal drinking ≥21 units (8.7%) | 6,014 | 1.48  (1.01, 2.15) | 0.61  (.30, 1.24) | 0.76  (.48, 1.20) | .22 | 5,757 | 1.42  (1.00, 2.08) | 0.65  (.36, 1.20) | 0.71  (.40, 1.16) | .27 |
| Partner drinking 4+ units *everyday^1^* (5.2%) | 5,359 | 0.90  (.44, 1.82) | 0.84  (.30, 2.38) | 1.15  (.70, 1.90) | .40 | 5,146 | 0.84  (.42, 1.70) | 0.90  (.44, 1.80) | 1.18  (.60, 2.35) | .37 |

*Note: ^1^Maternal reports of partner’s alcohol consumption; Model 1 adjusted for maternal age at delivery, parity, social economic position, maternal education, maternal smoking during first trimester in pregnancy, housing tenure, income, and maternal depressive symptoms at 32 weeks gestation; Model 2 further adjusted for maternal alcohol use at 18 weeks gestation; CL: childhood limited, AO: adolescent onset, EOP: early onset persistent, the Low conduct problems class was used as the reference group*
