# Supplementary material for: Parental alcohol use and risk of behavioral and emotional problems in offspring
Source: PLoS One. 2017 Jun 6;12(6):e0178862. doi: 10.1371/journal.pone.0178862 (PMC5460848; doi:10.1371/journal.pone.0178862)
Supplement: S9 Table — (A) Parental alcohol use (assessed at age 4 and 12 years using linear alcohol measures) and adolescent depressive symptoms–unweighted estimates-complete cases. Note. 1Maternal reports of partner’s alcohol consumption; Model 1 adjusted for maternal age at delivery, parity, social economic position, maternal education, maternal smoking during first trimester in pregnancy, housing tenure, income, and maternal depressive symptoms at 32 weeks gestation; Model 2 further adjusted for maternal alcohol use at 18 weeks gestation. (B). Heavy parental alcohol use (assessed at ages 4 and 12 years using binary alcohol measures) and adolescent offspring depressive symptoms–unweighted estimates. Note. 1Maternal reports of partner’s alcohol consumption; Model 1 adjusted for maternal age at delivery, parity, social economic position, maternal education, maternal smoking during first trimester in pregnancy, housing tenure, income, and maternal depressive symptoms at 32 weeks gestation; Model 2 further adjusted for maternal alcohol use at 18 weeks gestation. (DOCX) [file pone.0178862.s011.docx]

*Table S9a.* Parental alcohol use (assessed at age 4 and 12 years using linear alcohol measures) and adolescent depressive symptoms – unweighted estimates - complete cases

|  |  |  | Model 1 | | | |  | Model 2 | | | | | | |
| --- | --- | --- | --- | --- | --- | --- | --- | --- | --- | --- | --- | --- | --- | --- |
|  |  | Intercept | |  | Slope |  | |  | Intercept |  | Slope |  | |  |
|  | *N* | *b* (95% CI) | | *p* | *b* (95% CI) | *p* | | *N* | *b* (95% CI) | *p* | *b* (95% CI) | *p* | |  |
| **Age 4 years** |  |  | |  |  |  | |  |  |  |  |  | |  |
| Maternal alcohol use in units - linear term | 4,837 | .001 (-.01, .01) | | .90 | .003 (-.02, .03) | .84 | | 4,638 | -.001 (-.01, .01) | .81 | .000 (-.03, .03) | | .99 |  |
| Partner drinking 4+ units^1^ – linear term | 4,335 | -.068 (-.13, -.01) | | .03 | .077 (-.11, .26) | .41 | | 4,171 | -.068 (-.13, -.01) | .03 | .043 (-.14, .23) | | .65 |  |
| **Age 12 years** |  |  | |  |  |  | |  |  |  |  | |  |  |
| Maternal alcohol use in units - linear term | 4,133 | .002 (-.01, .01) | | .64 | .022 (-.00, .05) | .06 | | 3,965 | .000 (-.01, .01) | .95 | .018 (-.01, .04) | | .13 |  |
| Partner drinking 4+ units – linear term^1^ | 3,901 | .026 (-.03, .09) | | .40 | .048 (-.13, .22) | .59 | | 3,758 | .028 (-.03, .09) | .37 | -.074 (-.11, .25) | | .42 |  |

*Note. ^1^Maternal reports of partner’s alcohol consumption; Model 1 adjusted for maternal age at delivery, parity, social economic position, maternal education, maternal smoking during first trimester in pregnancy, housing tenure, income, and maternal depressive symptoms at 32 weeks gestation; Model 2 further adjusted for maternal alcohol use at 18 weeks gestation*

*Table S9b.* Heavy parental alcohol use (assessed at ages 4 and 12 years using binary alcohol measures) and adolescent offspring depressive symptoms – unweighted estimates

|  |  |  | Model 1 | | | |  | Model 2 | | | | | | |
| --- | --- | --- | --- | --- | --- | --- | --- | --- | --- | --- | --- | --- | --- | --- |
|  |  | Intercept | |  | Slope |  | |  | Intercept |  | Slope |  | |  |
|  | *N* | *b* (95% CI) | | *p* | *b* (95% CI) | *p* | | *N* | *b* (95% CI) | *p* | *b* (95% CI) | *p* | |  |
| **Age 4 years** |  |  | |  |  |  | |  |  |  |  |  | |  |
| Maternal drinking ≥21 units (9.1%) | 4,837 | .024 (-.25, .30) | | .86 | .264 (-.58, 1.11) | .54 | | 4,638 | .005 (-.28, .29) | .97 | .250 (-.63, 1.13) | | .58 |  |
| Partner drinking 4+ units everyday^1^ (5.2%) | 4,335 | -.067 (-.48, .34) | | .75 | 1.06 (-.32, 2.44) | .13 | | 4,171 | -.087 (-.49, .32) | .68 | 1.102 (-.30, 2.50) | | .12 |  |
| **Age 12 years** |  |  | |  |  |  | |  |  |  |  | |  |  |
| Maternal drinking ≥21 units (14.9%) | 4,133 | -.004 (-.24, .24) | | .97 | . (-.11, 1.38) | .09 | | 3,965 | -.027 (-.28, .22) | .83 | .569 (-.19, 1.33) | | .14 |  |
| Partner drinking 4+ units everyday^1^ (7.4%) | 3,901 | .004 (-.37, .38) | | .98 | .801 (-.22, 1.82) | .13 | | 3,758 | .016 (-.36, .40) | .93 | .365 (-.66, 1.39) | | .49 |  |

*Note. ^1^Maternal reports of partner’s alcohol consumption; Model 1 adjusted for maternal age at delivery, parity, social economic position, maternal education, maternal smoking during first trimester in pregnancy, housing tenure, income, and maternal depressive symptoms at 32 weeks gestation; Model 2 further adjusted for maternal alcohol use at 18 weeks gestation*
